# The earliest evidence for a supraorbital salt gland in dinosaurs in new Early Cretaceous ornithurines

**DOI:** 10.1038/s41598-018-22412-8

**Published:** 2018-03-05

**Authors:** Xia Wang, Jiandong Huang, Yuanchao Hu, Xiaoyu Liu, Jennifer Peteya, Julia A. Clarke

**Affiliations:** 1 School of Biological Science and Technology, University of Jinan, Jinan, 250022 China; 2 Anhui Geological Museum, Hefei, Anhui 230031 China; 3 Department of Biology & Integrated BioScience Program, University of Akron, Akron, OH USA; 4 Department of Geological Sciences, Jackson School of Geoscience, University of Texas, Austin, TX 78712 USA

## Abstract

Supraorbital fossae occur when salt glands are well developed, a condition most pronounced in marine and desert-dwelling taxa in which salt regulation is key. Here, we report the first specimens from lacustrine environments of the Jehol Biota that preserve a distinct fossa above the orbit, where the salt gland fossa is positioned in living birds. The Early Cretaceous ornithurine bird specimens reported here are about 40 million years older than previously reported Late Cretaceous marine birds and represent the earliest described occurrence of the fossa. We find no evidence of avian salt gland fossae in phylogenetically earlier stem birds or non-avialan dinosaurs, even in those argued to be predominantly marine or desert dwelling. The apparent absence of this feature in more basal dinosaurs may indicate that it is only after miniaturization close to the origin of flight that excretory mechanisms were favored over exclusively renal mechanisms of salt regulation resulting in an increase in gland size leaving a bony trace. The ecology of ornithurine birds is more diverse than in other stem birds and may have included seasonal shifts in foraging range, or, the environments of some of the Jehol lakes may have included more pronounced periods of high salinity.

## Introduction

Salt glands in birds (*glandulae nasalis*^1^) produce hypertonic solutions (mostly NaCl or KCl in some herbivorous taxa) that are excreted to help maintain appropriate internal solute levels while minimizing water loss^2–4^. The presence of glands with a similar capability is widely distributed in reptiles including turtles, sea snakes, lizards, crocodiles and birds, and they have been considered possibly ancestral to that clade^5,6^. Despite their broad distribution, glands with this function are found in a distinct locations on the skull in these different taxa, but a supraorbital position is only known in birds^1,2^.

Within birds, osmotic balance is regulated both by the kidneys and by salt glands. Salt glands have been identified in at least 40 families, representing nearly all traditional orders of birds except the Passeriformes^6^. They are most common in marine birds (e.g. gulls, petrels, albatrosses, auks, and penguins), but also seen in some more freshwater species (e.g. dabbling ducks, mallards, rails^7^), desert dwelling taxa (e.g. Ostrich, North African partridge^6,8^) and in some carnivorous birds with high protein diet (e.g. Tawny Eagles^9^). Salt glands in birds are known to exhibit remarkable levels of phenotypic plasticity morphologically and physiologically^4,8,10,11^, but their size and excretory capacity are largely decided by habitat salinity and diet^12,13^.

**Figure 1:**
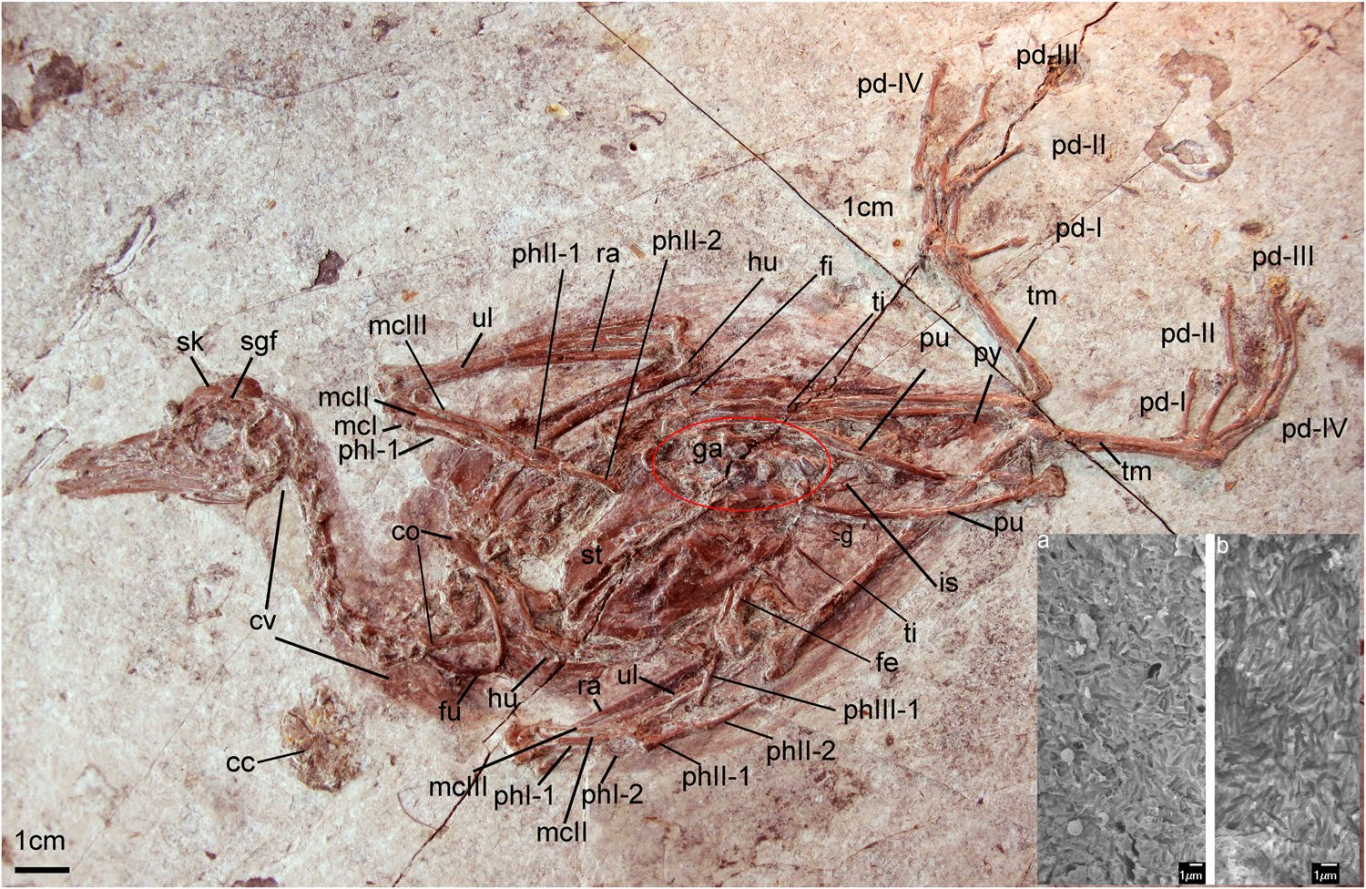
Photograph of the newly referred specimen AGB5841. Anatomical abbreviations: cc, crop content; co, coracoid; cv, cervical vertebra; fe, femur; fu, furcula; g, gastralia; ga, gastrolith; h, hyoid; hu, humerus; il, ilium; mcI–III, metacarpals I–III; pd I–IV, pedal digits I–IV; phI-1, the first phalanx of digit I; phI-2, the second phalanx of digit I; phII-1,the first phalanx of digit II; phII-2,the second phalanx of digit II; pu, pubis; py, pygostyle; ra, radius; rad, radiale; ri, rib; sc, scapula; sgf, salt gland fossa; sk, skull;; ti, tibiotarsus; tm, tarsometatarsus; tv, thoracic vertebra; ul, ulna; uln, ulnare. Insets show melanosomes of sampled feathers of AGB5841. (**a**) Sampled from breast feather; (**b**) sampled from primary feather.

**Figure 2:**
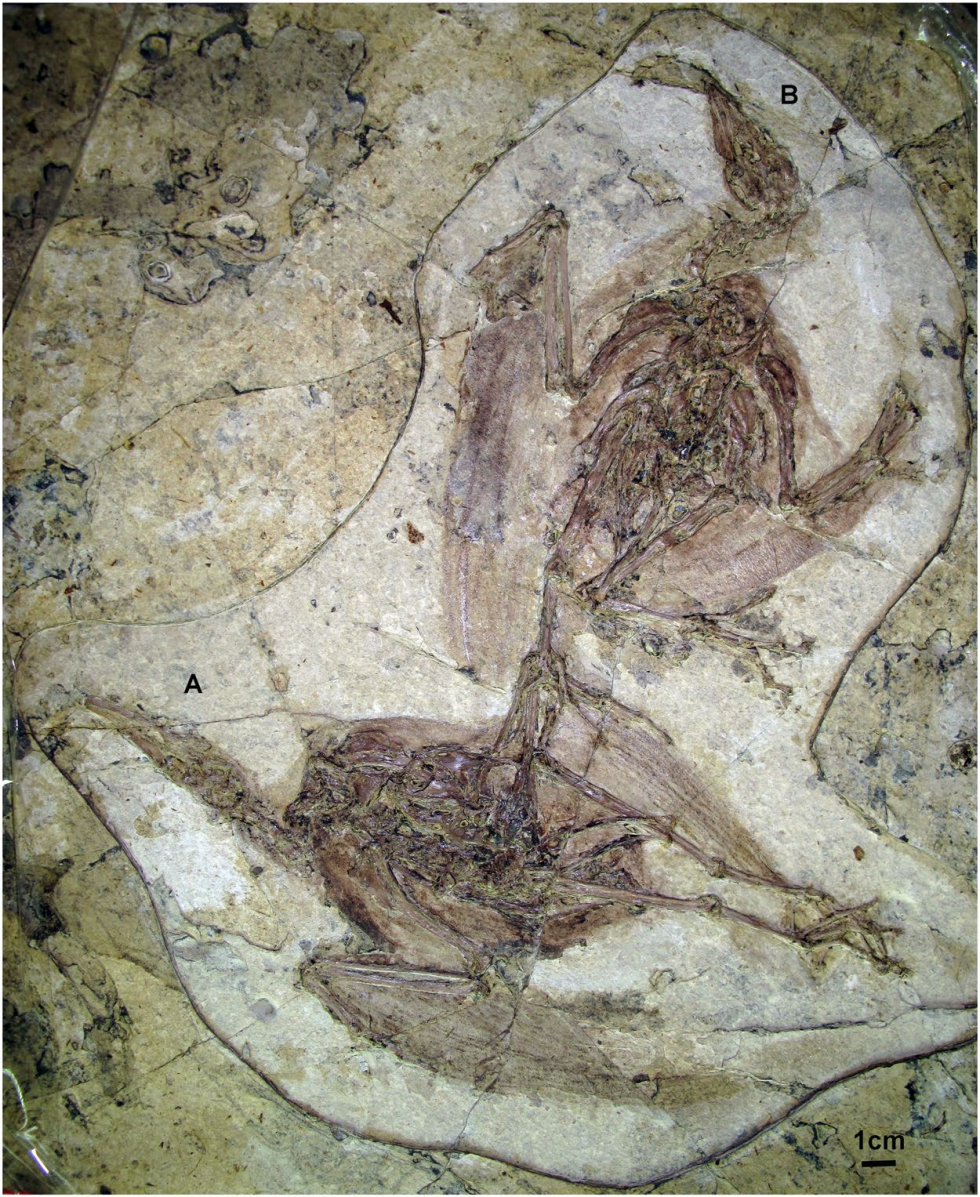
Photograph of the other two newly referred specimens (AGB5834-1/2).

In extinct birds, supraorbital salt gland fossae have only been reported in Late Cretaceous marine birds *Ichthyornis dispar* and the Hesperornithes (*Hesperornis regalis* and *Parahesperornis alexi*^14–16^) proposed to have a more tern-like and cormorant-like ecologies, respectively. To date, there has been no evidence for the presence of an avian supraorbital salt gland in non-avian dinosaurs or any other Mesozoic birds. The topic has received little attention, despite of the abundance of exceptional cranial materials available from the lacustrine environments of northeast China. The presence of nasal salt glands in predominantly ornithischian dinosaurs was proposed based on the size of the external nares^17^ but subsequent refuted^18^. Here, we report the presence of distinct salt gland fossa on the frontal of ornithurines birds from the Early Cretaceous Sihedang locality, which is near Lingyuan City, in western Liaoning, China (Jiufotang Formation; ^~^Aptian^19^). We estimate the phylogenetic relationships of these and other recently described Sihedang birds and review the evidence for the evolution of the salt gland in dinosaurs. So far the only other reported ornithurines from Sihedang locality are the *Gansus zheni holotype specimen*^20^ and the holotype of *Iteravis huchzermeyeri*^21^. Here we propose the new specimens (Figs 1 and 2) and the *Gansus zheni holotype specimen* are referable to the earlier named species, *Iteravis huchzermeyeri*^21^.

## Descriptioin and Comparison

### Skull

**Figure 3:**
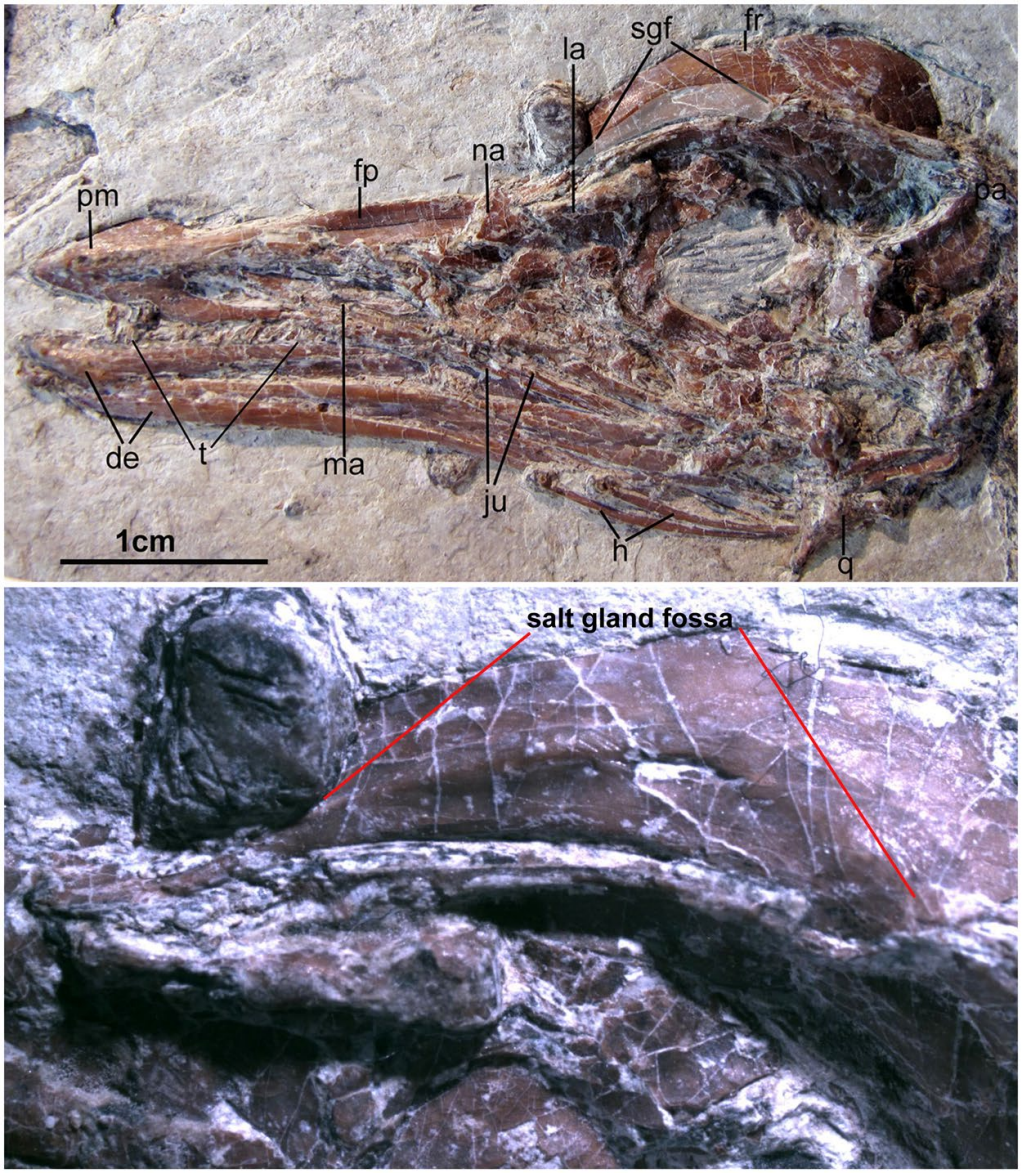
Photograph (top) and photomicrograph (bottom) of the skull. Anatomical abbreviations: de, dentary; fp, frontal process; fr, frontal; h, hyoid; ju, jugal; la, lacrimal; ma, maxilla; na, nasal; pa, parietal; pm, premaxilla; q, quadrate; sgf, salt gland fossa (the facet on the frontal for a supaorbital salt gland); t, tooth/teeth.

**Figure 4:**
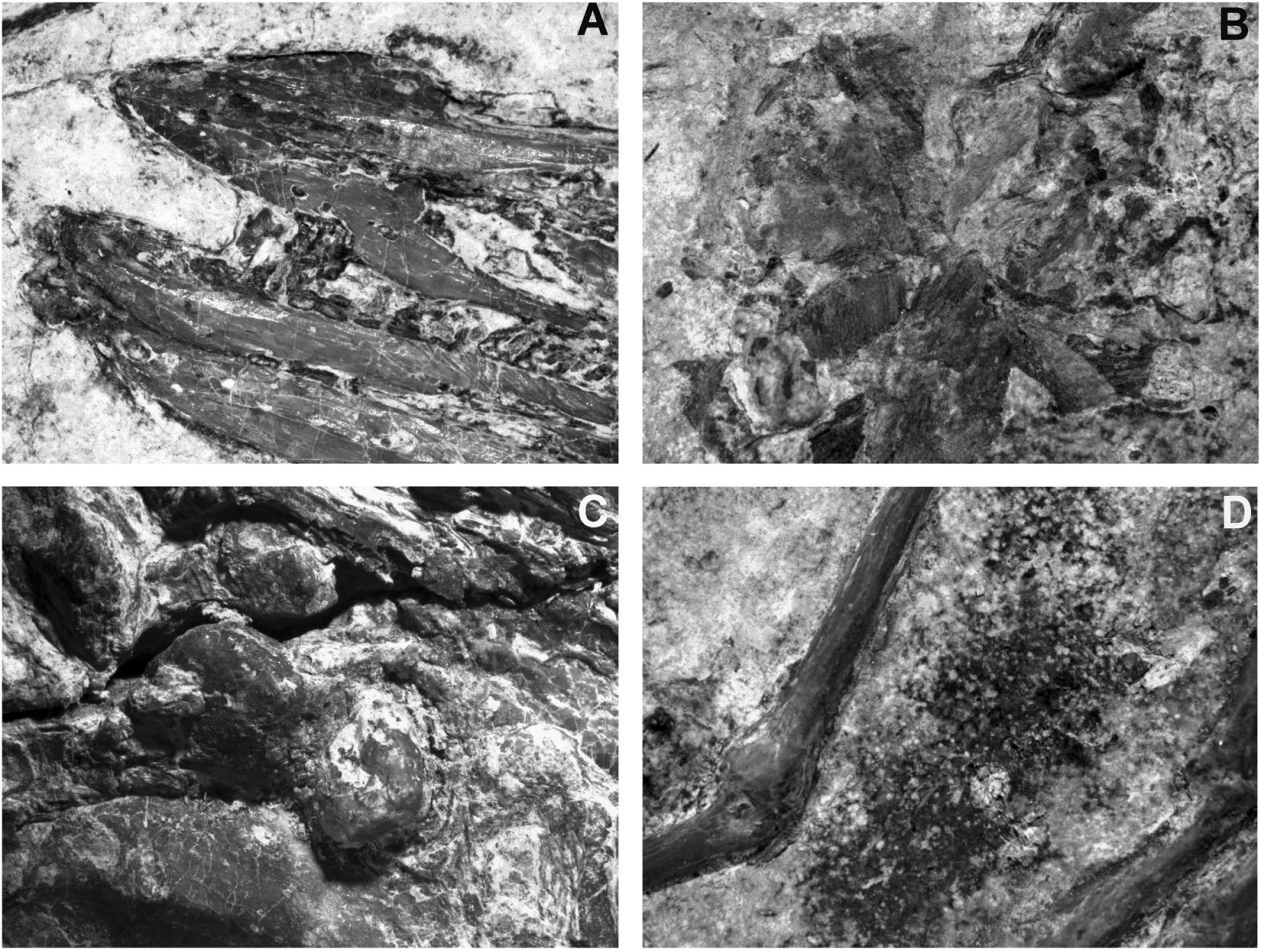
Anatomical details of specimen AGB5841. (**A**) Dentary teeth; (**B**) plant-like detritus cranial to pectoral girdle; (**C**) Gastroliths; (**D**) Close-up of soft-tissue preserved around toes.

The skull of AGB5841 (Fig. 3) is exposed in lateral view. It has a long, straight and robust rostrum and mandible, similar to that of *Yanornis martini*^22^, *Iteravis huchzermeyeri*^21^ and the *Gansus zheni* holotype^20^. The rostrum comprises about half of the total skull length (Fig. 3, Table 1), the same as *Yanornis martini*, *Iteravis huchzermeyeri* and *Gansus zheni*. A predentary bone is present (Fig. 3). The premaxillae taper cranially. Their dorsal processes contact the frontal. They are not fused to each other caudally. Mental foramina line the lower jaw, and the premaxilla bears several neurovascular pits. The maxilla appears makes up the majority of the facial margin. The dentary is forked caudally. No teeth appear preserved on the tip of dentary or on upper jaw (Figs 3 and 4A). However, preservation of the rostrum does not allow the dentition to be assessed with confidence. The premaxilla is toothless in both *Iteravis huchzermeyeri* and *Gansus zheni*, and in these two species, several teeth are visible on maxilla. In *Yanornis*, at least 9 teeth are present in the caudal premaxilla. The caudal part of dentary is packed with 12–16 socketed teeth. These teeth are small and lack serrations. Individual tooth crown morphology is well preserved in one displaced tooth. The crown is robust, conical in shape and slightly curved caudally. The caudal margin of the nares appears to be formed by the descending process of the nasal and a smaller dorsal process of the maxilla. Two fragments, one contacting the frontal, and the second displaced to near the base of the orbit, are interpreted as the remains of the lachrymal.

**Table 1:** Measurements of the new specimens (AGB5841, ABG 5834-1/2) and other published specimens (IVPP V18958^5^; BMNHC Ph1342, 1318^3^) (in cm) L/R. In this study we referred all these specimens to *Iteravis huchzermeyeri*.

	AGB5841	AGB5834-1	AGB5834-2	IVPP V18958	BMNHC-Ph1342	BMNHC Ph1318
Skull length	4.80			4.60	5.33	4.54
Premaxilla length along facial margin	1.35	—	—	1.56		
Dentary length, total; from anterior tip to fork of dorsal and ventral processes	3.40	4.19^*^	4.11^*^			
Dentary dorsoventral height at anterior tip	0.16	0.18	0.18			
Vertebral column						
Cervical vertebra average length	0.60	0.64	0.65			
Sacrum length (est.)	—	2.68	—			
Pectoral girdle						
Sternum length on midline	3.63	3.36	3.58			
Scapula maximum length, breadth just distal to glenoid facet	3.12^*^	—	3.71/4.17	3.50	4.04	
Coracoid height	2.26	2.13 L	2.39 L	2.10	2.07/2.07	2.29/2.12
Coracoid sternal margin length	1.44	1.45	1.50	1.50	1.60	1.50
Coracoidal lateral process length	0.57	0.35	0.26			
Furcula: length clavicular ramus (right)	1.78	1.57	1.28			
Pectoral limb						
Humerus maximum length	5.55 L	5.06/4.75	5.46/5.54	5.20	5.46/5.34	5.36/5.23
Radius length	5.51 L	5.53/5.09	5.38/4.86	4.9*		
Radius midshaft width	0.17	0.21/0.22	0.23/0.23			
Ulna length	5.9 L	5.3/5.25	5.40/5.38	5.30	5.61/5.51	5.41 R
Ulna midshaft width	0.36	0.37/0.35	0.32/0.33			
Carpometacarpus maximum length	2.62 L	2.52/2.59	2.63/2.67^*^	2.20	2.71/2.56	2.16 R
Metacarpal I length	0.68 L	0.41 R	0.58 R	0.40		
Metacarpal III width	0.09 L	0.10	0.07	0.10		
Metacarpal II width	0.19 L	0.18	0.18	0.20		
Phalanx length I:1	0.98 R	1.00	0.81	0.95		
Phalanx length I:2	0.48 R	0.27	0.29	0.40		
Phalanx II:1	1.48/1.25	1.12	1.28	1.15		
Phalanx II:2	1.07/1.1	1.01	1.00	1.10		
Phalanx II:3	0.26	0.30	0.28	0.30		
Phalanx III:1	0.53/0.54	0.48	0.53 L	0.60		
Pelvic girdle						
Ilium length total	4.10 L	—	—			
Ilium preacetabular	1.77	—	—			
Ischium length (estimated)	—	—	—			
Pubis length	4.76/4.81	—	—	4.10	4.87 L	4.91 L
Pubis average shaft diameter	0.19/0.25	—	—			
Pubis symphysis length	0.63	0.65	0.67			
Pelvic limb						
Femur maximum length	3.65 R	3.29 R	3.3/3.56	3.50	3.64 L	3.45/3.46
midshaft width	0.36	0.46	0.32			
Tibia maximum length, not including cnemial crest	6.55/6.67	5.94 L	6.4/6.31	5.90	6.44/6.61	6.38/6.54
Tarsometatarsus maximum length	3.41/3.67	3.3/3.71	3.49/3.57	3.10	3.79/3.81	3.69/3.65
Pedal phalanx I:1 length	0.86/0.77	—	0.64/0.58	0.80	0.84/0.82	0.77/0.75
Pedal phalanx II:1	1.3/1.37	0.98	1.13 R	1.1*	1.5/1.49	1.37/1.29
Pedal phalanx II:2	1.34/1.33	1.22 L	1.17	1.15	1.35/1.44	1.20/1.27
Pedal phalanx III:1	1.38/1.44	1.44	1.17	1.20	1.55/1.43	1.35/1.38
Pedal phalanx III:2	1.46/1.04	1.16	0.64	1.00	1.09/1.10	1.04/1.04
Pedal phalanx III:3	0.97/0.93	0.78	0.63	0.80	1.01/0.99	0.88/0.87
Pedal phalanx IV:1	1.05	1.50	1.01	1.00	1.06/1.10	1.04/0.98
Pedal phalanx IV:2	0.93/0.83	0.91	0.95	0.80	0.90/0.87	0.85/0.83
Pedal phalanx IV:3	0.83/0.84	0.95	0.85	0.80	0.85/0.85	0.80/0.83
Pedal phalanx IV:4	0.77/0.83	0.89	0.79^*^	0.70	0.82/0.83	0.79/0.71
Feathers						
Remiges: right side, maximum length distal primaries (9 & 8?)	9.31	13.92/10.38	13.5/9.36			

*Estimated; L, left; R, right.

The orbit is large with ring of scleral ossicles preserved. The mesethmoid creates an incomplete interorbital septum and contacts the frontal near the cranial margin of the orbit, as in *Yanornis martini*, *Iteravis huchzermeyeri* and *Gansus zheni*. On the craniodorsal frontal a distinct shallow fossa (Fig. 3) is developed in the location of a salt gland fossa in extant birds. The distinct shallow facet on the frontal of AGB5841 is shallower than in *Ichthyornis dispar* and *Hesperornis regalis* and similar to that of *Gansus zheni*. The frontal is slightly domed. The frontal/parietal suture appears to be open. The jugal is thin and rod-like and an ascending process does not appear present. The left quadrate has shifted out of position, and only the blocky otic process is visible with a lateral depression running along its length. Paired ceratobranchials as well as short epibranchials of the hyoid apparatus lie adjacent to the caudal left mandible. A spherical mass of fibrous, possibly vegetative material (Fig. 4B) is preserved near the pectoral girdle, anterior to the cervical vertebrae.

### Vertebral column

**Figure 5:**
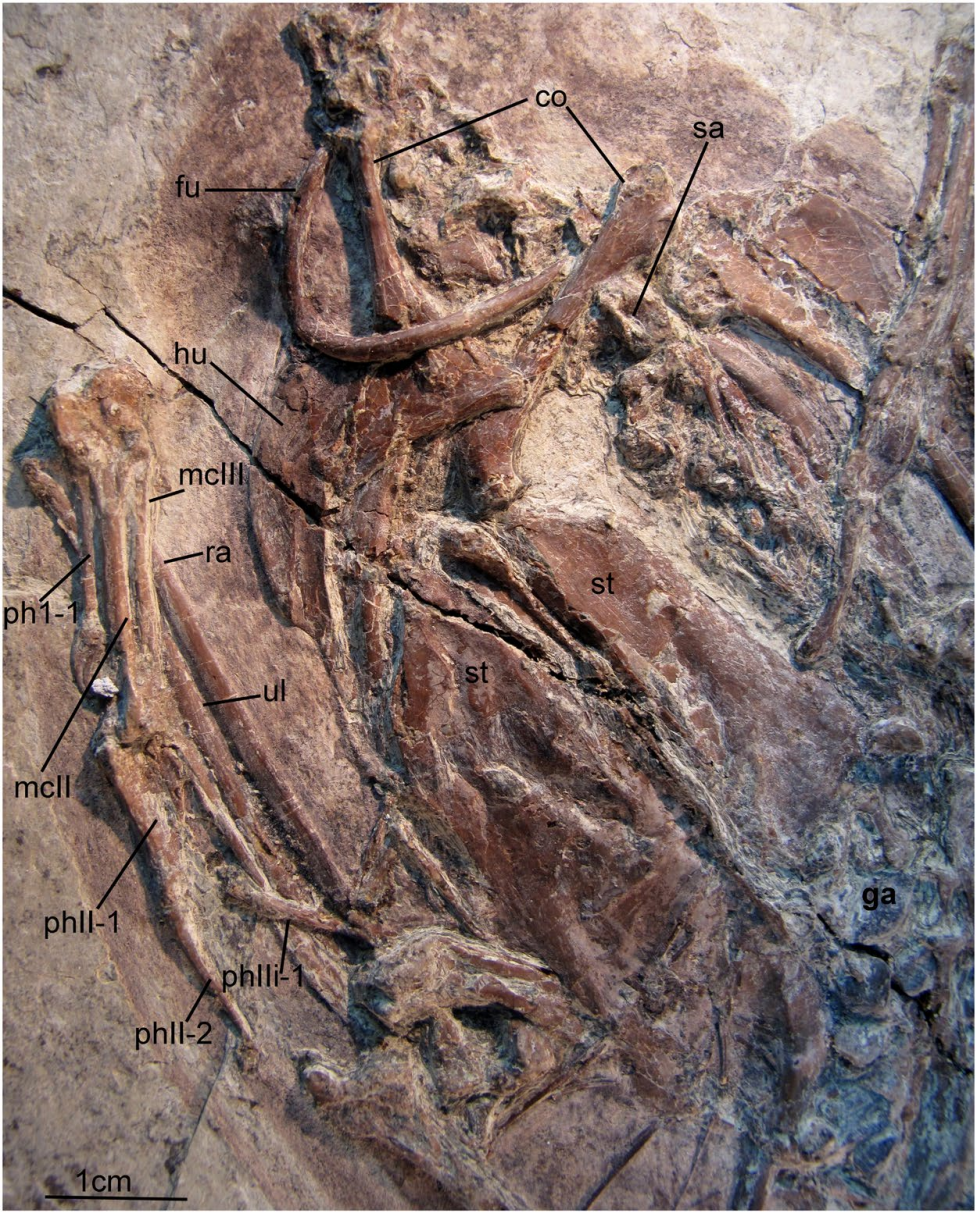
Thoracic girdle and limb of AGB5841.Abbreviations as in Fig. 1.

Eleven or twelve cervical vertebrae are present, similar to *Yanornis martini* (at least 10) and less than *Ichthyornis dispar* (14–16 cervical vertebrae). The fourth to ninth vertebrae are associated with reduced, spine-shaped pleurapophysis, which is about three quarters of vertebrae in length. Mid-series cervical vertebrae have a length about twice the width, with well-developed postzygopophyses. Thoracic vertebrae are relatively poorly exposed, but with deep lateral excavations are visible in the cranial -most three (AGB5841). The sacrum is composed of 9 to 10 vertebrae. There are 6 free caudal vertebrae and the pygostyle is short, flattened, about 3 anterior caudal vertebrates in length. Approximately 5 ribs are in articulation with the sternum. Sternal ribs are arranged as in extant birds; the ribs form an acute angle with the sternum. Uncinate process is present. A few gastralia present in the pubis area. A large cluster of gastroliths (Figs 4C and 5) lie in the abdominal areas of all three specimens. These appear to be sub-rounded to sub-angular quartz grains and have an average diameter of 2 mm. These stones show similarities with those in *Gansus yumenensis* and are proportionately larger and less numerous than other Jehol ornithuromorphs (e.g. *Archaeorhynchus spathula* and *Hongshanornis longicresta*).

### Pectoral girdle and limb

A keel runs the length of the sternum, which is exposed in ventral view (Fig. 5). A keel extending to the cranial margin of the sternum is present in ornithurines, (e.g. *Yixianornis grabaui*^22,23^, *Yanornis martini*^22^, *Iteravis huchzermeyeri*^21^ and *Gansus zheni*^20^. On the cranial sternal margin, coracoidal sulcus are visible. It seems no craniolateral processes preserved on any of these specimens. Caudally, a pair of large lateral processes with slightly expanded distal ends is present (also see in AGB5834-2, Fig. 2). It is unclear whether the medial processes met the sternal midline to enclose a fenestra as in *Iteravis huchzermeyeri*^24^, *Yanornis martini*, *Yixianornis grabaui*^22^ and *Gansus yumenensis*^25^. A small zyphoid processes is present in AGB5834-1 (Fig. 2). The right coracoid (Fig. 5) is preserved in ventral view, and the left one is in dorsal view. They are strut-like, with a small lateral process and a deep, round concave scapular facet (Fig. 5). Procoracoid process is not clear to see. The glenoid is situated sternal to the acrocoracoid process. The scapular blade is recurved and tapers distally. A long and pointed acromion is present. It appears to lack a projected apophysis.

The ulna is just slightly longer than the humerus. The left humerus (Fig. 1) is well preserved in cranial view. The deltopectoral crest of the humerus is approximately shaft-width in dorsal projection and exceeds just over one-third the length of the humeral shaft, the same as in *Iteravis huchzermeyeri* and *Gansus zheni*. The left ulna is exposed in dorsal view. Proximally, a prominent olacranon is developed, and the brachial impression is long and narrow. The distal dorsal condyle has a semilunate trochlear surface. Nine evenly spaced impressions for the attachment of the secondary feathers are visible along the caudal edge of ulna. The radius is narrow and approximately half the width of the ulna. It bears a prominent bicipital tubercle at the proximal end; the distal end of the radius is spoon-shaped.

Proximally, metacarpals I, II and III are fused to each other and to the semilunate carpal (Figs 1 and 5). Metacarpal I is straight and reaches the proximal terminus of the intermetacarpal space. It appears not fused to metacarpal II distally, which is also seen in *Yanornis martini* (IVPP V13358). It completely fused to metacarpal II in *Gansus yumenensis* and *Icththyornis dispar*^16^. Metacarpals II and III are fused to each other distally and subequal in distal extent. The craniocaudal diameter of Metacarpal III is less than half that of II and intermetacarpal space between them are very narrow. A small extensor process of the alular metacarpal is present as in *Iteravis huchzermeyeri*. The extensor groove extends straight down the ventral surface of metacarpal II, with a slightly projected scar for the distal retinacular restraint near its distal terminus. This projected scar is not as large as in *Ichthyornis dispar*, where this feature is developed as a pronounced tubercle^16^. Phalanx I.1 is straight and less than half the length of the carpometacarpus, more reduced than *Yixianornis grabaui*, where Phalanx I.1 is bowed and extremely elongated^23^. Phalanx II.1 is distinctively expanded craniocaudally, the second phalanx is slender and slightly shorter than the first phalanx. The third digit retains only the first phalanx, which is small and has a slightly expanded proximal end. A similar tubercle on the caudal margin of minor digit phalanx is known in *Yumenornis huangi*, *Gansus yumenensis*, *Iteravis huchzermeyeri*, *Gansus zheni*, AGB5841, *Ichthyornis dispar* and crown birds. Reduced claws are retained in the first two digits.

### Pelvic girdle and limb

**Figure 6:**
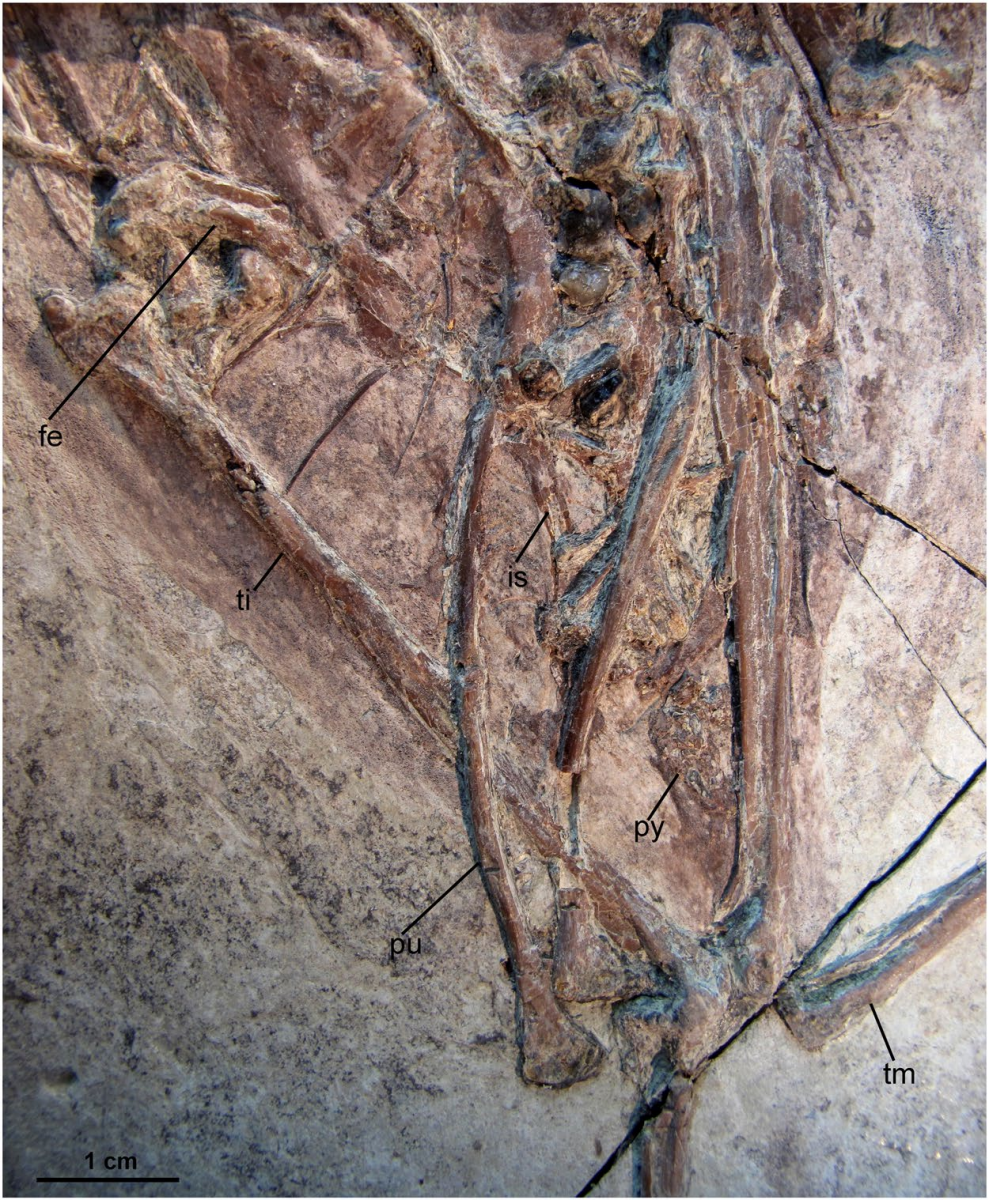
Pelvic girdle and limb of AGB5841.Abbreviations as in Fig. 1.

The pelvic girdle is preserved in life position in referred specimen AGB5834-2 (Fig. 2). The ilium is fused with the synsacrum. The pre-acetabular portion of the ilium is about the same length of post-acetabular portion. The pubes are rod-like and retroverted, not parallel to the Ischia (Fig. 6). Their distalmost are in contact but not fused and expanded into a boot like structure with a long symphysis, which pelsiomorphic for the theropods and also can be seen in *Hongshanornis longicresta*, *Yanornis martini*, *Schizooura lii*, *Iteravis huchzermeyeri* and *Gansus zheni*. The ischium is much shorter than the pubis. It tapers caudally and appears to expand slightly caudally into a small distally placed dorsal process (Fig. 6), as seen in *Yanornis martini*, *Gansus yumenensis*, *Gansus zheni*, *Piscivoravis lii and Iteravis huchzermeyeri*.

The femur and tarsometatarsi are relatively short, with femur slightly bow-shaped and shorter than the tarsometatarsi, unlike the condition in *Yixianornis grabaui* and *Yanornis martini*. The *Piscivoravis lii* tibiotarsus is elongated with a ratio of the femur length 1.8, similar to *Iteravis huchzermeyeri* (1.7) and *Gansus zheni* (1.9), higher than that of *Yanornis martini* (1.5). The fibula is about half the length of the tibiotarsus, whereas in *Yanornis martini*, fibular is reduced to less than the half. The left tibiotarsus is exposed in lateral view. The right tibiotarsus is in cranial view with distal end twisted in cranial-lateral view. It possesses projected rounded cnemial crests (Fig. 6). The distal condyles of the tibia are separated by an intercondylar depression. The fibula is reduced and runs along about 60% of the total length of tibiotarsus, similar to that of *Iteravis huchzermeyeri* and *Gansus zheni*. While in *Yanornis martini*, fibula length is less than half of that of tibiotarsus, in *Gansus yumenensis*, the fibula is unreduced and terminates proximal to the ankle^22,25^.

The tarsometatarsus is about half the length of the tibiotarsus (Figs 1 and 2; Table 1). The distal tarsals are fused to the metatarsals, and the metatarsals are co-osified proximally and distally to enclose a distal vascular foramen. Metatarsal V is not present. Metatarsal I swung back to the plantar surface but does not appear to be conspicuously twisted as in *Gansus yumenensis*. Metatarsal III is extended furthest distally, metatarsal IV is just slightly shorter than III, and metatarsal II is the shortest. Metatarsal II exhibits plantar deflection. The phalanges are long and slender with well-formed distal ginglymous trochleae and distinct pits for the attachment of the collateral ligaments. Pedal digits are robust compared with *Gansus yumenensis* and *Ichthyornis*. Pedal digit III is the most robust and longest with digit IV approaching it in length, which is comparable to *Yanornis martini* and *Yixianornis grabaui*. In *Gansus zheni*, digit IV is about the same or slightly longer than digit III (99–106%) and in *Iteravis huchzermeyeri* the ratio is 110%. In *Gansus yumenensis*, however, digit IV is much longer than III (110–122%). Digit II is slightly shorter than digits III and IV. Phalanx lengths decrease distally. All unguals are small, short with only the ungula on digit I weakly recurved. Flexor tubercules (Fig. 4D) on unguals are weakly developed as in *Yanornis martini*, *Gansus zheni* and *Iteravis huchzermeyeri*, not that pronounced and distal located as in *Gansus yumenensis*^25^. Impressions of soft-tissue which was covered in carbon films are preserved in lobed shape around toes (Figs 1 and 4D), similar to those reported in *Yanornis martini*^26^, whereas in *Gansus yumenensis*, tubercular impressions of soft-tissue preserved all around toes. Thus, lobed feet like those in coot and grebes were likely developed in AGB5841.

### Feathers

Feather remains are well preserved as impressions in all specimens (Figs 1 and S2), better preserved in AGB 5834-1, 2. Remnants of body contour feathers are associated with the cranium and the cervical area as well as near wing and leg elements. The primary feathers have a maximal length of 213 mm in AGB 5834-1, 2 and 105 mm in AGB 5841. The asymmetrical primary feathers are extremely elongated with narrow rachises, suggesting long and slender wings as seen in *Confuciusornis* and *Archaeorhynchus spathula*, much longer than those observed in *Yanornis martini* and *Gansus yumenensis*. The alula is not preserved. The tail feathers are not preserved. Scanning electron microscopy (SEM) results show that contour and primary feather samples contain melanosome molds^27,28^ that are closely spaced and elongate with rounded termini (Fig. 1). Their aspect ratio (length:width ratio:1.93–9.41) is typical of eumelanosomes seen in black feathers^27^.

### Salt gland fossa

**Figure 7:**
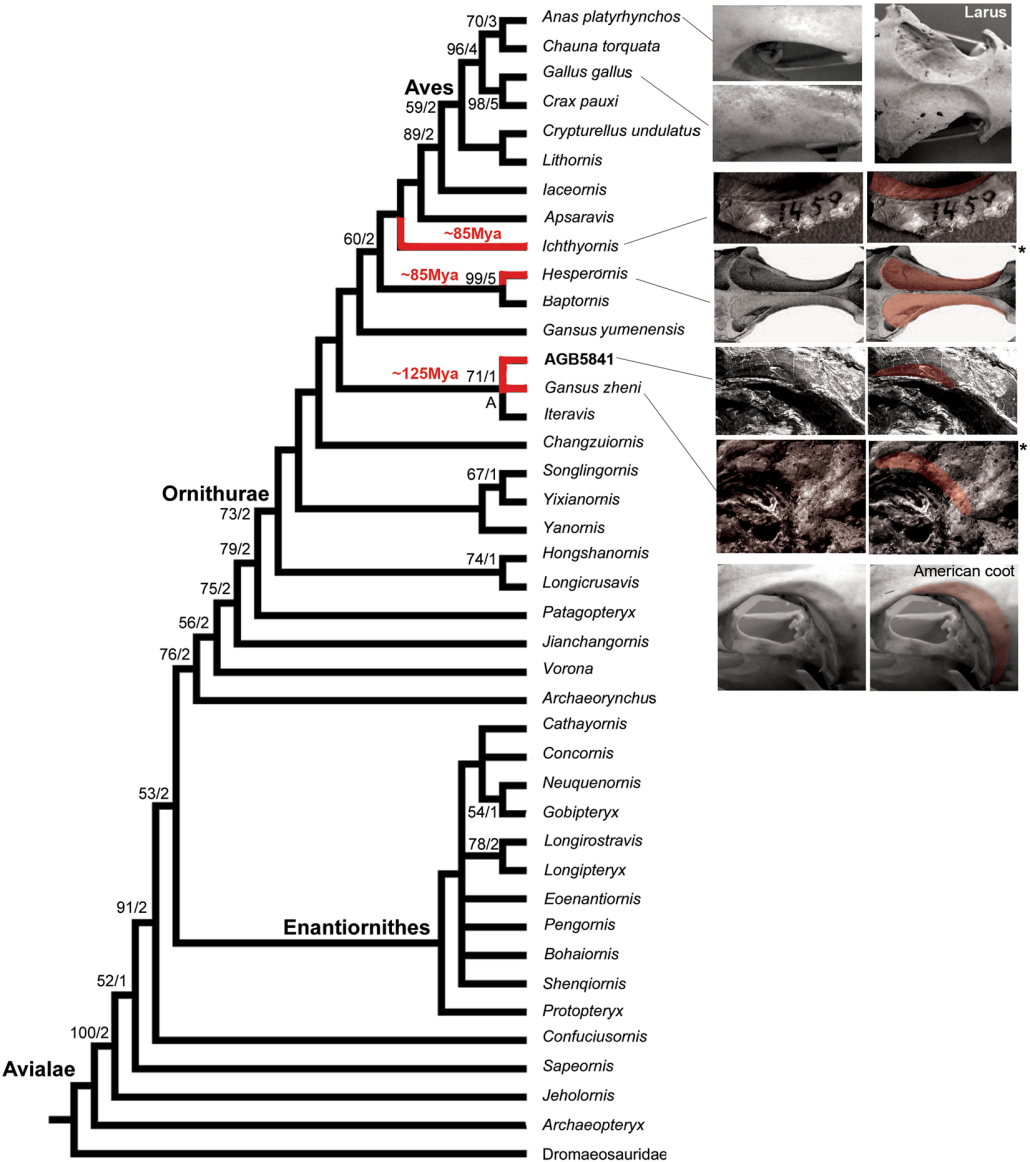
Strict consensus cladogram illustrating the phylogenetic position of AGB5841 and presence of salt gland fossa (with red shadows) along the phylogeny [length L: 590, CI: 0.50, RI 0.79, RC 0.39 (PIC only)]. Bootstrap support for those nodes recovered in greater than 50% of the 1000 replicates performed and Bremer (1988) support values are reported to the right of the node to which they apply (Format: Bootstrap/Bremer). *Images were modified from^14,21^ accordingly.

The shallow salt gland facet on the frontal of AGB5841 is edged by a slightly raised rim (cc s. 1, 2). This rim is more distinct rostrally and more faint caudally, similar to those seen in rails (e.g. *Fulica americana*; Fig. 7). We noted a similar facet in the *Gansus zheni* holotype specimen; in both specimens it is shallower and smaller than in *Ichthyornis dispar* and *Hesperornis*^14^ (Fig. 2). In these taxa, deep salt gland facets extend almost the full length of the frontals. The raised rims of these facets meet on the midline in *Hesperornis*^14^, in many penguins, and in other marine taxa.

### Phylogenetic Analysis

The strict consensus tree (Fig. 7) places AGB5841 as in a clade with *Gansus zheni* and *Iteravis huchzermeyeri*. The monophyly of this new clade (node A) of Chinese ornithurines, recovered with significant bootstrap support (71% of replicates) and is supported by three unambiguously optimized synapomorphies (numbers refer to characters and states listed in Appendix) including 170:0, distal end of pubis expanded, flared; 217:2, digit III almost equal to digit IV, digit II shortest; 221:1, presence of shallow salt gland fossa. *Gansus yumenensis*, the name bearing species of the taxon *Gansus*, is not recovered as part of this clade. The phylogenetic position of *Gansus zheni*, *Iteravis* and AGB5841 supports the assignment of both *Gansus zheni* and the new specimens to *Iteravis huchzermeyeri*.

## Discussion

The new specimens are similar to *Iteravis huchzermeyeri*^21^ and *Gansus zheni*^20^ in both size and morphology. They share with these taxa a unique combination of characters (e.g. elongated rostrum; tubercle on posterior margin of minor digit phalanx; elongate and toothless premaxilla, U-shaped furcula, pubis with an expanded distal boot, ischium with weak dorsal process at midpoint, pedal digit III subequal to digit IV, digit II shortest; preservation of gastroliths (Table 1, Fig. 4). Furthermore, the salt gland facet preserved on the anterodorsal frontal in AGB5841 can also be seen in *Gansus zheni*^20^ (Figs 2 and 7). The frontal is poorly preserved in *Iteravis huchzermeyeri*, preventing determination that whether the “salt gland fossa” is developed or not. In sum, given the shared characters, comparable size, and provenance from the same locality, Sihedang, it is likely that *Iteravis huchzermeyeri* and *Gansus zheni* are synonyms and together with AGB5841 represent one species. This result is also supported by their resolution as a clade in phylogenetic analysis (Fig. 7). As *Iteravis huchzermeyeri* was the first published name, it would be the valid species name for these specimens. *Iteravis huchzermeyeri* shares characters with *Gansus yumenensis* but differs from *Yanornis martini* in possessing more gracile furcular rami, a manual digit I slightly shorter and manual digit I:2 smaller, more proximally projecting cnemial crests, relatively more elongate tibia, an ischium with a dorsal process, more gracile tarsometatarsus and elongate pedal digits with elongate proximal phalanges (lacking in *Yanornis*). It is similar to *Yanornis martini* but differs from *Gansus yumenensis* in possessing a more elongated rostrum, a metatarsal III longer than IV, the length of digit III is subequal to IV, a reduced fibula and the claws of pedal digits III and IV lacking a prominent pendant flexor tubercle.

### Salt gland

The Early Cretaceous specimens (AGB5841, ca.125 mya) records the earliest evidence for a supraorbital salt gland fossa, 40my earlier than those known in Late Cretaceous ornithurine taxa. These previously reported salt gland fossae are all from taxa known from marine deposits (e.g. ref.^14^). Salt gland fossae have not been previously reported in avialan or non avialan dinosaurs from the lake deposits containing the Jehol Biota. Specifically, more than 16 species of ornithurine birds have been reported from approximately 12 Early Cretaceous localities in northern China, all from lacustrine deposits^24^. We reassessed these taxa and also could find no evidence of such fossae. We propose this feature may diagnose *Iteravis huchzermeyeri*. All specimens with salt gland fossae, AGB5841 and the *Gansus zheni and Iteravis huchzermeyeri* holotypes, are known from a single locality.

Habitat salinity and food type are the major factors in the relative development of salt glands. Environmental salinity plays a major role in determining the nature of the osmoregulatory challenge facing any organism^29^ and the principle role of the salt glands is to excrete excess sodium ions^4^. The presence of salt gland fossa in a single taxon from only one Jehol locality may be explained by environmental conditions. The lake sampled by the Sihedang locality may have showed meromictic interludes like those suggested for Lake Sihetun during the time period represented by the Jiufotang Formation^30^, which will episodically cause saline or semi-saline conditions^31^. Although this is not been previously proposed for the lake represented by the Sihedang locality, it is consistent with the idea that Jehol Biota lakes may represent a complex paleoenvironment. The fossiliferous deposits of Sihedang may sample specific conditions in the development of the lake system^21,32^.

The salt concentration of the diet is another important factor influencing the development of a salt gland and associated fossae^4^. Terrestrial carnivorous birds and freshwater birds (e.g. rails) that feed on invertebrates^2,7^ have a supraorbital salt gland in response to the sodium load from their food^33^. Taxa with piscivorous diet have been proposed to have less of an issue with salt balance than those that feed on invertebrates^2^.The gastroliths in *Iteravis huchzermeyeri* resemble those of *Gansus yumenensis* and *Yanornis martini*, but are larger and less numerous than those proposed herbivorous ornithuromorphs (e.g. *Archaeorhynchus spathula* and *Hongshanornis* longicresta), which may suggest a comparable lifestyle with *Gansus yumenensis* or *Yanornis martini*. The elongate rostrum, dentition, and the size and number of gastroliths and aquatic habitat imply that *Iteravis* might have been picivorous or a mixed feeder mainly on hard food, a diet unlikely to cause severe osmoregulation issues^2,29^. Other Jehol taxa with direct evidence of a picivorous diet do not show this feature (e.g. *Confuciusornis*^34^, *Yanorni*s^35^). It also might feed on invertebrates in fresh water as rails, given their similarities in habitat and salt gland fossa morphology. A difference in the behavior in this ornithurine species might be an alternative explanation for the presence of this feature. Although Sihedang was not closer to the paleocoastline than other Jehol sites^32^, *Iteravis* could conceivably have migrated to the sea in winter and returned in spring as is the case in some extant birds^36^.

Previous hypotheses concerning possible nasal salt gland in dinosaurs have relied primarily on correlations of salt glands with a variety of osteological characters, but no supraorbital salt glad fossae have been proposed to be present in any more basal dinosaur than *Iteravis*. Grooves, depressions or openings preserved in the narial region were originally proposed to be associated with nasal salt glands in some herbivorous dinosaurs (e.g. Ankylosaurs, hadrosaurines *Pinacosaurus grangeri*^37,38^, basal ornithischian *Hypsilophodon foxii* and sauropodomorph *Plateosaurus engelhardti*^17,39^. However, the location of the proposed gland (i.e. within the nasal vestibule, not external to the nasal cavity) is not seen in any extant archosaurs or sauropsids^18^ and a vascular function for the foramen in the external naris of *Pinacosaurus grangeri* was subsequently found more plausible^40^. The theory that herbivorous dinosaurs would use salt glands to excrete potassium ions^17^ is also not well supported; sodium chloride is the major electrolyte in extracellular body fluids of vertebrate animals and herbivores do not typically have major osmoregulation issues^41^.

It is uncertain if the supraorbital salt gland observed in orithurnine birds also characterizes a more inclusive clade of theropods. Gauthier^42^ suggested that a bird-like supraorbital gland may be present in *Troodon formosus*. However, further scrutiny of this character^43^ revealed that the proposed osteological correlates of a supraorbital gland were lacking. A supraorbital salt gland and fossae might be expected particularly in marine and desert-dwelling basal dinosaurs. However, there is no evidence for the presence of supraorbital facets in spinosaurid dinosaurs or *Confuciusornis* which have been proposed to be picivores (e.g. refs^34,44,45^) or in filter-feeding ornithomimid dinosaurs^43,46^. Witmer^18^ inferred that canals leading to a supraorbital salt gland may be present in *Allosaurus fragilis* and some tyrannosaurids given that lacrimal in these taxa bears a caudal foramen, near the prefrontal articulation. No distinct supraorbital facet was observed. While it is unclear why the ecology of these taxa of all theropods, would lead to evolution of a salt gland, these hypotheses merit further investigation.

The supraorbital position for a salt gland, and the differential development of an associated fossa in some orniturine taxa, may be a feature that evolved after the origin of flight as birds exploited novel ecologies enabled by this novel locomotor mode. Basal dinosaurs were mainly terrestrial forms proposed to have a comparatively low sodium intake^41^, so they may have relied on the kidney alone for osmoregulation. A combination of sustained small body size^47^ and dietary diversification may have led to novel osmoregulatory pressures in birds. The elimination of salts may be well within the capacity of the kidneys of larger theropods. Alternatively, a salt gland in more basal dinosaurs could be present but very small or differently located (e.g. within nasal cavity). More and better preserved fossils are needed to more fully illuminate the evolution of the supraorbital salt gland and its associated osmoregulatory function in dinosaurs.

### Gastroliths

So far, gastroliths have been reported in Yanornis martini, Archaeorhynchus spathula, Hongshanornis longicresta, Bohaiornis guoi, Iteravis and Gansus zheni, among them, Archaeorhynchus and Hongshanornis are fully edentulous^20,21,48–51^. The preservation of both fish remains (IVPP V 13259) and gastroliths (IVPP V 13358) in the abdominal region of Yanornis martini and was considered to have switched their diet from piscivorous to herbivorous^35^. Though with one more specimen (STM9-51) being found preserved a few gastroliths, a recent study, however, reinterpreted the stones reported in Yanornis martini as accidentally ingested sand based on their smaller size, greater number, size range, and more caudal location in the abdominal region. The diet of Yanornis was consequently reconstructed as primarily piscivorous^52^.

The three new specimen reported here are all preserve geo-gastroliths, with those in AGB5834 are similar to those of *Yanornis* in shape, number, size and large area occupied, which offers more support to the interpretation in ref.^35^. It should further be noted that the interpretive drawing of the alimentary canal of *Yanornis* in ref.^52^ is not accurate; the ventriculus (gizzard) is shown more dorsally and cranially located than its natural position in living birds and where the stomach was depicted is mostly occupied by the reproductive organs; the grit is not too caudally located to represent gizzard stones but in the right position as in living birds. Analyses of skeleton morphology and claw curvature reconstructed *Yanornis* as more ground-foraging, which is consistent with the gastrolith evidence^35^; Moreover, ground feeders tend to be more likely to accumulate large amount of gastroliths compared to aerial or arboreal feeders^53^, which would explain the large quantity of stones in *Yanornis*. The great variation of size (diameter from less than 0.2 mm to 2.7 mm) and shape (subrounded or angular) of gastroliths in *Yanornis* and the new specimens are consistent with experimental data that gizzard stones in herbivorous birds experienced fast abrasion, but no significant rounding or polish developed^54^. We suggest that although we can’t rule out that these stones were ingested accidently, the interpretation of grit preserved as gastroliths is consistent and consequently diet-switching in *Yanornis* and related taxa is tenable.

## Conclusions

New specimens referred to *Iteravis huchzermeyeri* suggest that *Gansus zheni* is a junior synonym of this species. Supraorbital salt gland fossae are only so far known in one species from one locality in the Jehol system. While development of this feature may be partially explained by local environmental conditions (saline periods in lake development) or even behavioural differences, other widespread basal avialan taxa with a similar proposed diet (e.g. *Confuciusornis*) do not show a distinct facet. The new specimens reported here represent both the phylogenetically earliest occurrence of this feature and its first Early Cretaceous record. While a supraorbital salt gland seen in Aves may likely have an origin predating Ornithurae, earlier proposed evidence is ambiguous and does not include extant avian signatures such as development of a distinct facet. Body size constraints and novel feeding ecologies associated with the evolution of flight may drive the presence of a supraorbital salt gland in dinosaurs. However, new fossil evidence is needed to further investigate the evolution of novel osmoregulatory mechanisms in dinosaurs and their potentially distinct osteological signatures.

## Materials and Methods

The holotype specimen of *Iteravis huchzermeyeri*^21^ is IVPP V18958 (Institute of Vertebrate Paleontology and Paleoanthropology) is a nearly complete, articulated specimen with feather impressions^21^. Here we refer to this species: AGB5841 (Anhui Geological Museum), a nearly complete specimen with feather impressions (Fig. 1), AGB5834-1,-2, two complete individuals in one slab (Fig. S1), BMNHC-Ph1342, 1318 (Beijing Natural History Museum, China), articulated skeletons preserved in ventral view (holotype and referred specimen of *Gansus zheni*^20^.

We investigated the phylogenetic position of the new specimens using the best-preserved specimen, AGB5841, as an exemplar and assessing a dataset modified from that of^51^. This dataset was revised by modifying and ordering one character (relative length of pedal digits) and adding 5 new Ornithurae^55,56^, *Gansus yumenensis*, *Gansus zheni*, *Iteravis huchzermeyeri*,*Changzuiornis ahgm* and AGB5841 (Appendix I,II). One new character (presence of salt gland fossa) was added. Forty-one taxa were evaluated for a total of 221 morphological characters. All analyses were performed using PAUP 4.0b10^57^ using a maximum parsimony estimator. Heuristic searches were used given the size of the taxonomic sample. Three thousand replicates of random stepwise addition (branch swapping: tree-bisection-reconnection) were performed holding only one tree at each step. Branches were collapsed to create polytomies if the minimum branch length was equal to zero. One thousand bootstrap replicates with ten random stepwise addition heuristic searches per replicate were also performed with the same settings as in the primary analysis. Bootstrap support for those nodes recovered in greater than 50 percent of the 500 replicates performed and Bremer support values are reported to the right of the node to which they apply (Format, Bootstrap/Bremer). Bremer support values were calculated by iterative searches for suboptimal trees in PAUP 4.0b10 using the same heuristic search strategy as the primary analysis. 15 most parsimonious trees (MPTs) were recovered (L = 582, CI = 0.50, RI = 0.80, RC = 0.40; PIC only).
