# Author Correction: The earliest evidence for a supraorbital salt gland in dinosaurs in new Early Cretaceous ornithurines

**DOI:** 10.1038/s41598-020-80628-z

**Published:** 2021-01-12

**Authors:** Xia Wang, Jiandong Huang, Yuanchao Hu, Xiaoyu Liu, Jennifer Peteya, Julia A. Clarke

**Affiliations:** 1 School of Biological Science and Technology, University of Jinan, Jinan, 250022 China; 2 Anhui Geological Museum, Hefei, 230031 Anhui China; 3 Department of Biology & Integrated BioScience Program, University of Akron, Akron, OH USA; 4 Department of Geological Sciences, Jackson School of Geoscience, University of Texas, Austin, TX 78712 USA

Correction to: *Scientific Reports*, 10.1038/s41598-018-22412-8, published online 05 March 2018

The Supplementary Information files are missing from this Article. The Supplementary Information files appear below.

## Supplementary Information

Supplementary Information.

Appendix I.

Appendix II.

