# Supplementary material for: Author Correction: The earliest evidence for a supraorbital salt gland in dinosaurs in new Early Cretaceous ornithurines
Source: Sci Rep. 2021 Jan 12;11:1730. doi: 10.1038/s41598-020-80628-z (PMC7803944; doi:10.1038/s41598-020-80628-z)
Supplement: Supplementary file 1 — Supplementary Information. [file 41598_2020_80628_MOESM1_ESM.pdf]

Supporting Information

The earliest evidence for a supraorbital salt gland in dinosaurs in new Early Cretaceous ornithurines

Included:

1. Description (Figure S1-S3)
2. Gastroliths
3. Table S1 Measurements

1. Supplemental description of the new specimens

Skull

The skull of AGB5841 (Fig. 1) is exposed in lateral view. It has a long, straight and robust rostrum and mandible, similar to that of *Yanornis*[1], *Iteravis huchzermeyeri*[2] and the *Gansus zheni* holotype [3]. The rostrum comprises about half of the total skull length (Fig. 1, Fig.S1), the same as *Yanornis*, *Iteravis* and *Gansus zheni*. A predentary bone is present (Fig. 1). The premaxillae taper cranially. Their dorsal processes contact the frontal. They are not fused to each other caudally. Mental foramina line the lower jaw, and the premaxilla bears several neurovascular pits. The maxilla appears makes up the majority of the facial margin. The dentary is forked caudally. No teeth appear preserved on the tip of dentary or on upper jaw (Fig. 1, Fig.S1). However, preservation of the rostrum does not allow the dentition to be assessed with confidence. The premaxilla is toothless in both *Iteravis* and *Gansus zheni*, and in these two species, several teeth are visible on maxilla. In *Yanornis*, at least 9 teeth are present in the caudal premaxilla. The caudal part of dentary is packed with 12-16

socketed teeth. These teeth are small and lack serrations. Individual tooth crown morphology is well preserved in one displaced tooth. The crown is robust, conical in shape and slightly curved caudally. The caudal margin of the nares appears to be formed by the descending process of the nasal and a smaller dorsal process of the maxilla. Two fragments, one contacting the frontal, and the second displaced to near the base of the orbit, are interpreted as the remains of the lachrymal.

The orbit is large with ring of scleral ossicles preserved. The mesethmoid creates an incomplete interorbital septum and contacts the frontal near the cranial margin of the orbit, as in *Yanornis*, *Iteravis* and *Gansus zheni*. On the craniodorsal frontal a distinct shallow fossa (Fig.S2A) is developed in the location of a salt gland fossa in extant birds. The distinct shallow facet on the frontal of AGB5841 is shallower than in *Ichthyornis* and *Hesperornis* and similar to that of *Gansus zheni*. The frontal is slightly domed. The frontal/parietal suture appears to be open. The jugal is thin and rod-like and an ascending process does not appear present. The left quadrate has shifted out of position, and only the blocky otic process is visible with a lateral depression running along its length. Paired ceratobranchials as well as short epibranchials of the hyoid apparatus lie adjacent to the caudal left mandible. A spherical mass of fibrous, possibly vegetative material (Fig.S2B) is preserved near the pectoral girdle, anterior to the cervical vertebrae.

Vertebral column

Eleven or twelve cervical vertebrae are present, similar to *Yanornis* (at least 10) and less than *Ichthyornis* (14-16 cervical vertebrae). The fourth to ninth vertebrae are associated with reduced, spine-shaped pleurapophysis, which is about three quarters of vertebrae in length. Mid-series cervical vertebrae have a length about twice the width, with well-developed postzygopophyses. Thoracic vertebrae are relatively poorly exposed, but with deep lateral excavations are visible in the cranial -most three (AGB5841). The sacrum is composed of 9 to 10 vertebrae. There are 6 free caudal vertebrae and the pygostyle is short, flattened, about 3 anterior caudal vertebrate in length. Approximately 5 ribs are in articulation with the sternum. Sternal ribs are arranged as in extant birds; the ribs form an acute angle with the sternum. Uncinate process is present. A few gastralia present in the pubis area. A large cluster of gastroliths (Fig.S2C) lie in the abdominal areas of all three specimens. These appear to be sub-rounded to sub-angular quartz grains and have an average diameter of 2 mm. These stones show similarities with those in *Gansus* and are proportionately larger and less numerous than other Jehol ornithuromorphs (e.g. *Archaeorhynchus* and *Hongshanornis*).

Pectoral girdle and limb

A keel runs the length of the sternum, which is exposed in ventral view (Fig. 1). A keel extending to the cranial margin of the sternum is present in ornithurines, (e.g. *Yixianornis*[1, 4], *Yanornis*[1], *Iteravis*[2] and *Gansus zheni*[3]. On the cranial sternal margin, coracoidal sulcus are visible. It seems no craniolateral processes preserved on any of

these specimens. Caudally, a pair of large lateral processes with slightly expanded distal ends is present (also see in AGB5834-2, Fig. S1). It is unclear whether the medial processes met the sternal midline to enclose a fenestra as in *Iteravis*[5], *Yanornis*, *Yixianornis*[1] and *Gansus yumenensis*[6]. A small zyphoid processes is present in AGB5834-1 (Fig. S1). The right coracoid (Fig. 1A) is preserved in ventral view, and the left one is in dorsal view. They are strut-like, with a small lateral process and a deep, round concave scapular facet (Fig. S1). Procoracoid process is not clear to see. The glenoid is situated sternal to the acrocoracoid process. The scapular blade is recurved and tapers distally. A long and pointed acromion is present. It appears to lack a projected apophysis.

The ulna is just slightly longer than the humerus. The left humerus (Fig. 1) is well preserved in cranial view. The deltopectoral crest of the humerus is approximately shaft-width in dorsal projection and exceeds just over one-third the length of the humeral shaft, the same as in *Iteravis* and *Gansus zheni*. The left ulna is exposed in dorsal view. Proximally, a prominent olacranon is developed, and the brachial impression is long and narrow. The distal dorsal condyle has a semilunate trochlear surface. Nine evenly spaced impressions for the attachment of the secondary feathers are visible along the caudal edge of ulna. The radius is narrow and approximately half the width of the ulna. It bears a prominent bicipital tubercle at the proximal end; the distal end of the radius is spoon-shaped.

Proximally, metacarpals I, II and III are fused to each other and to the semilunate

carpal (Fig. 1A). Metacarpal I is straight and reaches the proximal terminus of the intermetacarpal space. It appears not fused to metacarpal II distally, which is also seen in *Yanornis martini* (IVPP V13358). It completely fused to metacarpal II in *Gansus yumenensis* and *Icththyornis*[7]. Metacarpals II and III are fused to each other distally and subequal in distal extent. The craniocaudal diameter of Metacarpal II is less than half that of III and intermetacarpal space between them are very narrow. A small extensor process of the alular metacarpal is present as in *Iteravis*. The extensor groove extends straight down the ventral surface of metacarpal II, with a slightly projected scar for the distal retinacular restraint near its distal terminus. This projected scar is not as large as in *Ichthyornis*, where this feature is developed as a pronounced tubercle[7]. Phalanx I.1 is straight and less than half the length of the carpometacarpus, more reduced than *Yixianornis*, where Phalanx I.1 is bowed and extremely elongated[4]. Phalanx II.1 is distinctively expanded craniocaudally, the second phalanx is slender and slightly shorter than the first phalanx. The third digit retains only the first phalanx, which is small and has a slightly expanded proximal end. A similar tubercle on the caudal margin of minor digit phalanx is known in *Yumenornis huangi, Gansus yumenensis*, *Iteravis huchzermeyeri*, *Gansus zheni*, AGB5841, *Ichthyornis* and crown birds. Reduced claws are retained in the first two digits.

Pelvic girdle and limb

The pelvic girdle is preserved in life position in referred specimen AGB5834-2

(Fig.S1). The ilium is fused with the synsacrum. The pre-acetabular portion of the ilium is about the same length of post-acetabular portion. The pubes are rod-like and retroverted, not parallel to the Ischia (Fig. 1). Their distalmost are in contact but not fused and expanded into a boot like structure with a long symphysis, which pelsiomorphic for the theropods and also can be seen in *Hongshanornis, Yanornis, Schizooura, Iteravis* and *Gansus zheni*. The ischium is much shorter than the pubis. It tapers caudally and appears to expand slightly caudally into a small distally placed dorsal process (Fig. 1), as seen in *Yanornis*, *Gansus yumenensis*, *Gansus zheni, Piscivoravis and Iteravis*.

The femur and tarsometatarsi are relatively short, with femur slightly bow-shaped and shorter than the tarsometatarsi, unlike the condition in *Yixianornis* and *Yanornis*. The tibiotarsus is elongated with a ratio of the femur length 1.8, similar to *Iteravis* (1.7) and *Gansus zheni* (1.9), higher than that of *Yanornis* (1.5). The fibula is about half the length of the tibiotarsus, whereas in *Yanornis*, fibular is reduced to less than the half. The left tibiotarsus is exposed in lateral view. The right tibiotarsus is in cranial view with distal end twisted in cranial-lateral view. It possesses projected rounded cnemial crests (Fig. 1). The distal condyles of the tibia are separated by an intercondylar depression. The fibula is reduced and runs along about 60% of the total length of tibiotarsus, similar to that of *Iteravis* and *Gansus zheni*. While in *Yanornis*, fibula length is less than half of that of tibiotarsus, in *Gansus yumenensis*, the fibula is unreduced and terminates proximal to the ankle[6, 8].

The tarsometatarsus is about half the length of the tibiotarsus (Fig. 1). The distal tarsals are fused to the metatarsals, and the metatarsals are co-osified proximally and distally to enclose a distal vascular foramen. Metatarsal V is not present. Metatarsal I swung back to the plantar surface but does not appear to be conspicuously twisted as in *Gansus yumenensis*. Metatarsal III is extended furthest distally, metatarsal IV is just slightly shorter than III, and metatarsal II is the shortest. Metatarsal II exhibits plantar deflection. The phalanges are long and slender with well-formed distal ginglymous trochleae and distinct pits for the attachment of the collateral ligaments. Pedal digits are robust compared with *Gansus yumenensis* and *Ichthyornis*. Pedal digit III is the most robust and longest with digit IV approaching it in length, which is comparable to *Yanornis* and *Yixianornis*. In *Gansus zheni*, digit IV is about the same or slightly longer than digit III (99-106%) and in Iteravis the ratio is 110%. In *Gansus yumenensis*, however, digit IV is much longer than III (110-122%). Digit II is slightly shorter than digits III and IV. Phalanx lengths decrease distally. All unguals are small, short with only the ungula on digit I weakly recurved. Flexor tubercules (Fig. S2H) on unguals are weakly developed as in *Yanornis, Gansus zheni* and *Iteravis*, not that pronounced and distal located as in *Gansus yumenensis*[6]. Impressions of soft-tissue which was covered in carbon films are preserved in lobed shape around toes (Figs. S2H ,I), similar to those reported in *Yanornis* [9], whereas in *Gansus yumenensis*, tubercular impressions of soft-tissue preserved all around toes. Thus, lobed feet like those in coot and grebes were likely developed in AGB5841.

Feathers

Feather remains are well preserved as impressions in all specimens (Figs. 1, S2), better preserved in AGB 5834-1, 2. Remnants of body contour feathers are associated with the cranium and the cervical area as well as near wing and leg elements. The primary feathers have a maximal length of 213 mm in AGB 5834-1, 2 and 105mm in AGB 5841. The asymmetrical primary feathers are extremely elongated with narrow rachises, suggesting long and slender wings as seen in *Confuciusornis* and *Archaeorhynchus spathula*, much longer than those observed in *Yanornis martini* and *Gansus yumenensis*. The alula is not preserved. The tail feathers are not preserved. Scanning electron microscopy (SEM) results show that contour and primary feather samples contain melanosome molds[10, 11] that are closely spaced and elongate with rounded termini (Fig.S3). Their aspect ratio (length:width ratio:1.93-9.41) is typical of eumelanosomes seen in black feathers[10].

2. Gastroliths

So far, gastroliths have been reported in *Yanornis martini, Archaeorhynchus spathula*, *Hongshanornis longicresta*, *Bohaiornis guoi*, *Iteravis* and *Gansus zheni*, among them, *Archaeorhynchus* and *Hongshanornis* are fully edentulous[2, 3, 12-15]. The preservation of both fish remains (IVPP V 13259) and gastroliths (IVPP V 13358) in the abdominal region of *Yanornis martini* and was considered to have switched their diet from piscivorous to herbivorous[16]. A recent study, however, reinterpreted the stones

reported in *Yanornis martini* as accidentally ingested sand based on their smaller size, greater number, size range, and more caudal location in the abdominal region. The diet of *Yanornis* was consequently reconstructed as primarily piscivorous[17].

The three new specimen reported here are all preserve geo-gastroliths, with those in AGB5834 are similar to those of *Yanornis* in shape, number, size and large area occupied, which offers more support to the interpretation in[16]. It should further be noted that the interpretive drawing of the alimentary canal of *Yanornis* in[17] is not accurate; the ventriculus (gizzard) is shown more dorsally and cranially located than its natural position in living birds and where the stomach was depicted is mostly occupied by the reproductive organs; the grit is not too caudally located to represent gizzard stones but in the right position as in living birds. Analyses of skeleton morphology and claw curvature reconstructed *Yanornis* as more ground-foraging, which is consistent with the gastrolith evidence[16]; Moreover, ground feeders tend to be more likely to accumulate large amount of gastroliths compared to aerial or arboreal feeders[18], which would explain the large quantity of stones in *Yanornis*. The great variation of size (diameter from less than 0.2mm to 2.7mm) and shape (subrounded or angular) of gastroliths in *Yanornis* and the new specimens are consistent with experimental data that gizzard stones in herbivorous birds experienced fast abrasion, but no significant rounding or polish developed[19]. We suggest that although we can't rule out that these stones were ingested accidently, the interpretation of grit preserved as gastroliths is consistent and consequently diet-

switching in *Yanornis* and related taxa is tenable.

References

1. Zhou, Z. & Zhang, F. Two new ornithurine birds from the Early Cretaceous of western Liaoning, China. *科学通报(英文版)* **46**, 1258-1264 (2001).

2. Zhou, S., O'Connor, J.K. & Wang, M. A new species from an ornithuromorph (Aves: Ornithothoraces) dominated locality of the Jehol Biota. *Chinese Science Bulletin* **59**, 5366-5378 (2014).

3. Liu, D. et al. An advanced, new long-legged bird from the Early Cretaceous of the Jehol Group (northeastern China): insights into the temporal divergence of modern birds. *Zootaxa* **3884**, 253-266 (2014).

4. Clarke, J.A., Zhou, Z. & Zhang, F. Insight into the evolution of avian flight from a new clade of Early Cretaceous ornithurines from China and the morphology of Yixianornis grabaui. *Journal of anatomy* **208**, 287-308 (2006).

5. Zhou, Z. The Jehol Biota, an Early Cretaceous terrestrial Lagerstätte: new discoveries and implications. *National Science Review* **1**, 543-559 (2014).

6. You, H.-l. et al. A Nearly Modern Amphibious Bird from the Early Cretaceous of Northwestern China. *Science* **312**, 1640-1643 (2006).

7. Clarke, J.A. Morphology, phylogenetic taxonomy, and systematics of Ichthyornis and Apatornis (Avialae: Ornithurae). *Bulletin of the American Museum of Natural History*, 1-179 (2004).

8. Zhou, Z. & Zhang, F. Two new ornithurine birds from the Early Cretaceous of western Liaoning, China. *Chinese Science Bulletin* **46**, 1258-1264 (2001).

9. Zheng, X. et al. Fossil evidence of avian crops from the Early Cretaceous of China. *Proceedings of the National Academy of Sciences* **108**, 15904-15907 (2011).

10. Vinther, J., Briggs, D.E., Clarke, J., Mayr, G. & Prum, R.O. Structural coloration in a fossil feather. *Biology Letters* **6**, 128 (2010).

11. Li, Q. et al. Plumage color patterns of an extinct dinosaur. *Science* **327**, 1369 (2010).

12. Zhou, Z. & Zhang, F. A beaked basal ornithurine bird (Aves, Ornithurae) from the Lower Cretaceous of China. *Zoologica Scripta* **35**, 363-373 (2006).

13. O'Connor, J.K., Gao, K.-Q. & Chiappe, L.M. A new ornithuromorph (Aves: Ornithothoraces) bird from the Jehol Group indicative of higher-level diversity. *Journal of Vertebrate Paleontology* **30**, 311-321 (2010).

14. Chiappe, L.M. et al. A new specimen of the Early Cretaceous bird Hongshanornis longicresta: insights into the aerodynamics and diet of a basal ornithuromorph. *PeerJ* **2**, e234 (2014).

15. Li, Z., Zhou, Z., Wang, M. & Clarke, J.A. A new specimen of large-bodied basal enantiornithine Bohaiornis from the Early Cretaceous of China and the inference of feeding ecology in Mesozoic birds. *Journal of Paleontology* **88**, 99-108 (2014).

16. Zhou, Z., Clarke, J., Zhang, F. & Wings, O. Gastroliths in Yanornis: an indication of the earliest radical diet-switching and gizzard plasticity in the lineage leading to living birds? *Naturwissenschaften* **91**, 571-574 (2004).

17. Zheng, X. et al. New specimens of Yanornis indicate a piscivorous diet and modern alimentary canal. *Plos One* **9**, e95036 (2014).

18. Gionfriddo, J.P. & Best, L.B. Grit-use patterns in North American birds: the influence of diet, body size, and gender. *The Wilson Bulletin*, 685-696 (1996).

19. Wings, O. & Sander, P.M. No gastric mill in sauropod dinosaurs: new evidence from analysis of gastrolith mass and function in ostriches. *Proceedings of the Royal Society B: Biological Sciences* **274**, 635-640 (2007).

Table S1 Measurements of the new specimens (AGB5841, ABG 5834-1/2) and other published specimens (IVPP V18958[5]; BMNHC Ph1342, 1318[3]) (in cm) L/R. In this study we referred all these specimens to *Iteravis huchzermeyeri*.

	AGB5841	AGB5834-1	AGB5834-2	IVPP V18958	BMNHC-Ph1342	BMNHC Ph1318
Skull length	4.80			4.60	5.33	4.54
Premaxilla length along facial margin	1.35	-	-	1.56		
Dentary length, total; from anterior tip	3.40	4.19*	4.11*			
Dentary dorsoventral height at	0.16	0.18	0.18			
Vertebral column						
Cervical vertebra average length	0.60	0.64	0.65			
Sacrum length (est.)	-	2.68	-			
Pectoral girdle						
Sternum length on midline	3.63	3.36	3.58			
Scapula maximum length, breadth	3.12*	-	3.71/4.17	3.50	4.04	
Coracoid height	2.26	2.13L	2.39L	2.10	2.07/2.07	2.29/2.1
Coracoid sternal margin length	1.44	1.45	1.50	1.50	1.60	1.50
Coracoidal lateral process length	0.57	0.35	0.26			
Furcula: length clavicular ramus	1.78	1.57	1.28			
Pectoral limb						
Humerus maximum length	5.55L	5.06/4.75	5.46/5.54	5.20	5.46/5.34	5.36/5.2
Radius length	5.51L	5.53/5.09	5.38/4.86	4.9*		
Radius midshaft width	0.17	0.21/0.22	0.23/0.23			
Ulna length	5.9L	5.3/5.25	5.40/5.38	5.30	5.61/5.51	5.41R
Ulna midshaft width	0.36	0.37/0.35	0.32/0.33			
Carpometacarpus maximum length	2.62L	2.52/2.59	2.63/2.67*	2.20	2.71/2.56	2.16R
Metacarpal I length	0.68L	0.41R	0.58R	0.40		
Metacarpal III width	0.09L	0.10	0.07	0.10		
Metacarpal II width	0.19L	0.18	0.18	0.20		
Phalanx length I:1	0.98R	1.00	0.81	0.95		
Phalanx length I:2	0.48R	0.27	0.29	0.40		
Phalanx II:1	1.48/1.25	1.12	1.28	1.15		

Phalanx II:2	1.07/1.1	1.01	1.00	1.10		
Phalanx II:3	0.26	0.30	0.28	0.30		
Phalanx III:1	0.53/0.54	0.48	0.53L	0.60		
Pelvic girdle						
Ilium length total	4.10L	-	-			
Ilium preacetabular	1.77	-	-			
Ischium length (estimated)	-	-	-			
Pubis length	4.76/4.81	-	-	4.10	4.87L	4.91L
Pubis average shaft diameter	0.19/0.25	-	-			
Pubis symphysis length	0.63	0.65	0.67			
Pelvic limb						
Femur maximum length	3.65R	3.29 R	3.3/3.56	3.50	3.64L	3.45/3.4
midshaft width	0.36	0.46	0.32			
Tibia maximum length, not including	6.55/6.67	5.94L	6.4/6.31	5.90	6.44/6.61	6.38/6.5
Tarsometatarsus maximum length	3.41/3.67	3.3/3.71	3.49/3.57	3.10	3.79/3.81	3.69/3.6
Pedal phalanx I:1 length	0.86/0.77	-	0.64/0.58	0.80	0.84/0.82	0.77/0.7
Pedal phalanx II:1	1.3/1.37	0.98	1.13R	1.1*	1.5/1.49	1.37/1.2
Pedal phalanx II:2	1.34/1.33	1.22L	1.17	1.15	1.35/1.44	1.20/1.2
Pedal phalanx III:1	1.38/1.44	1.44	1.17	1.20	1.55/1.43	1.35/1.3
Pedal phalanx III:2	1.46/1.04	1.16	0.64	1.00	1.09/1.10	1.04/1.0
Pedal phalanx III:3	0.97/0.93	0.78	0.63	0.80	1.01/0.99	0.88/0.8
Pedal phalanx IV:1	1.05	1.50	1.01	1.00	1.06/1.10	1.04/0.9
Pedal phalanx IV:2	0.93/0.83	0.91	0.95	0.80	0.90/0.87	0.85/0.8
Pedal phalanx IV:3	0.83/0.84	0.95	0.85	0.80	0.85/0.85	0.80/0.8
Pedal phalanx IV:4	0.77/0.83	0.89	0.79*	0.70	0.82/0.83	0.79/0.7
Feathers						
Remiges: right side, maximum length	9.31	13.92/10.38	13.5/9.36			

* estimated; L, left; R, right.

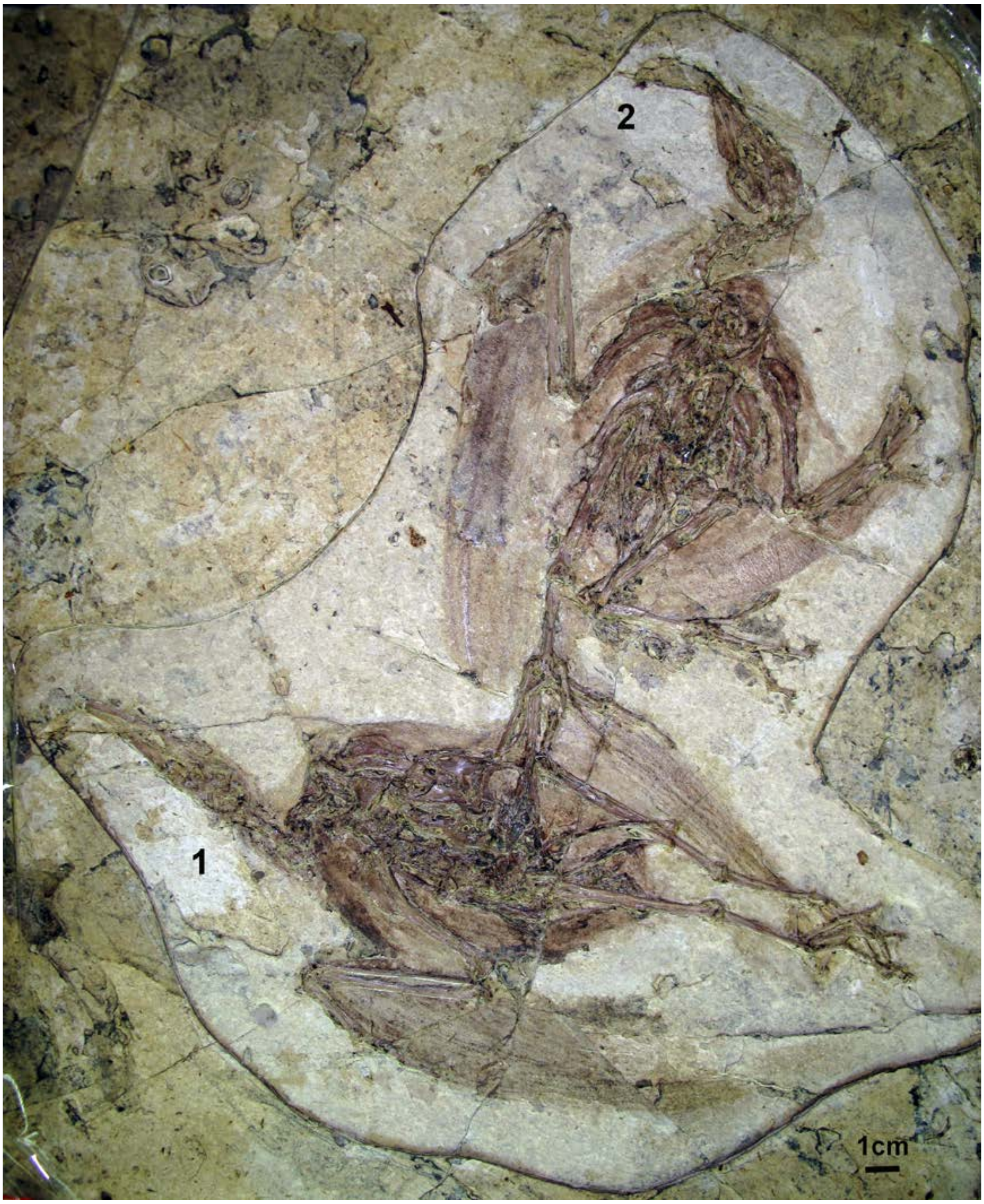

Figure S1. Photograph of the other two newly referred specimens (AGB5834-1/2).

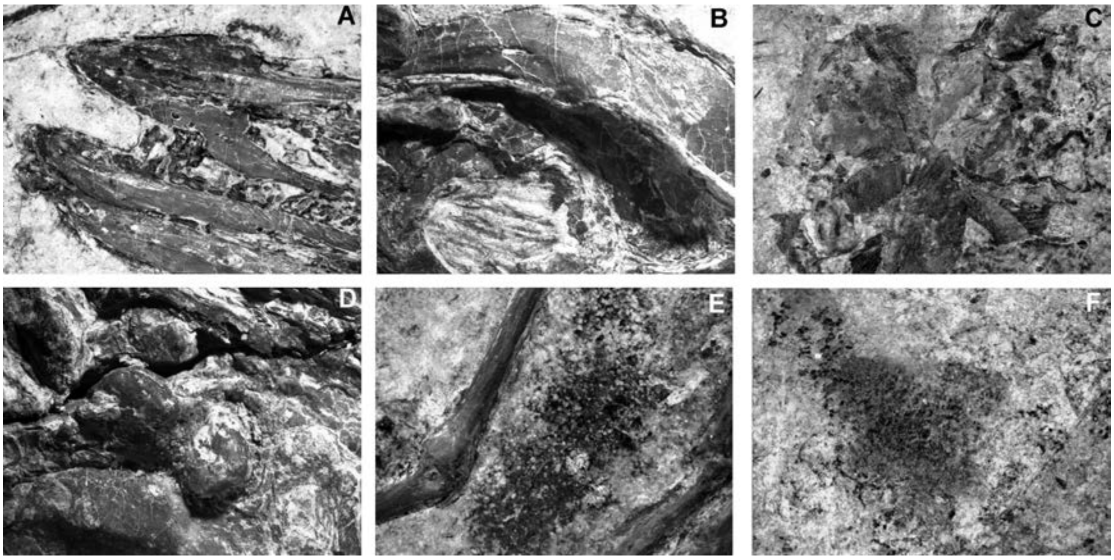

Figure S2. Anatomical details of specimen AGB5841. A, dentary teeth; B, Facet on the frontal for a supraorbital salt gland; C, plant-like detritus cranial to pectoral girdle; D, Gastroliths; E, F, Close-up of soft-tissue preserved around toes.

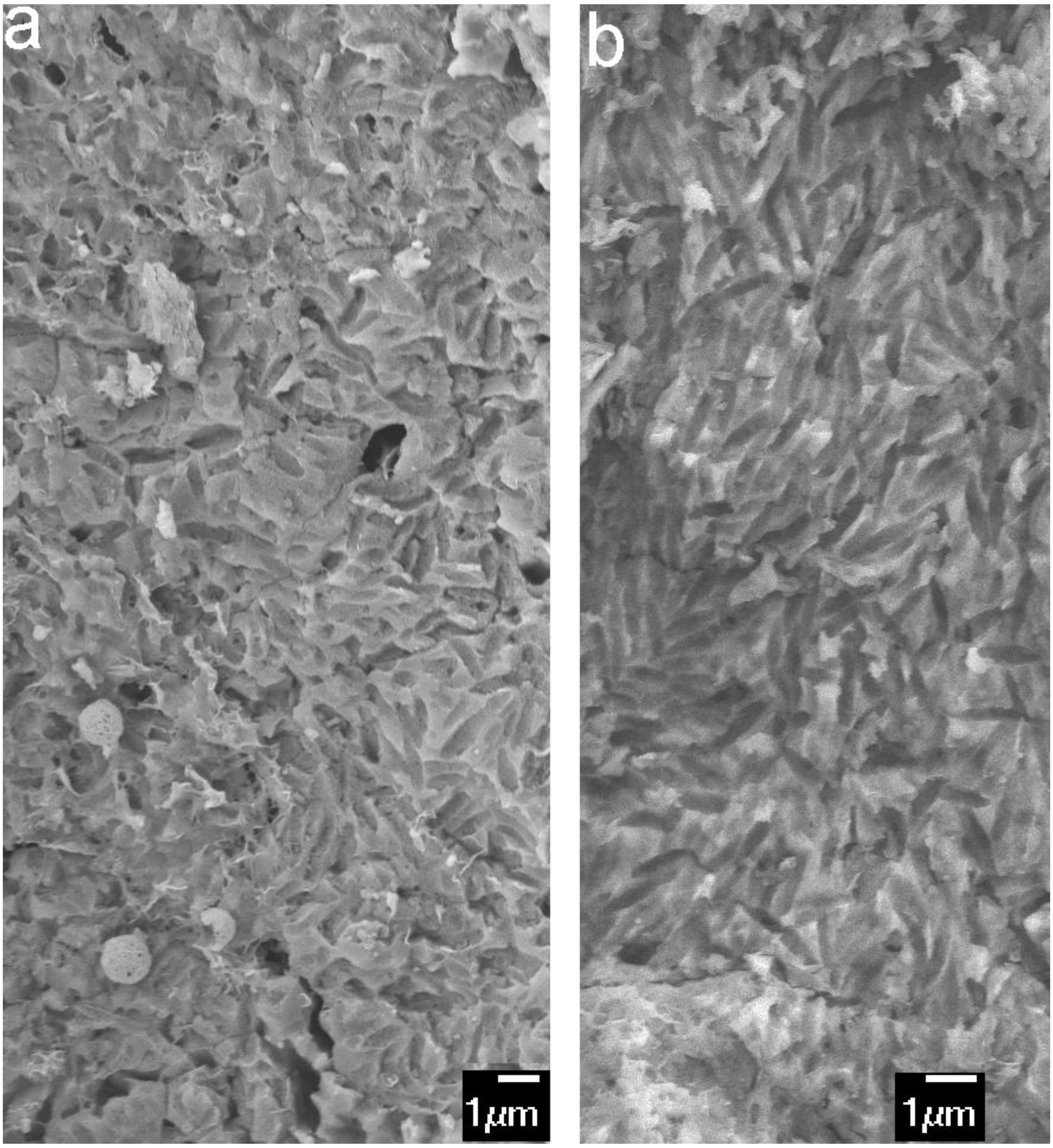

Figure S3. Melanosomes of sampled feathers of AGB5841. (a), sampled from breast feather; (b) sampled from primary feather.
