# Supplementary material for: Author Correction: The earliest evidence for a supraorbital salt gland in dinosaurs in new Early Cretaceous ornithurines
Source: Sci Rep. 2021 Jan 12;11:1730. doi: 10.1038/s41598-020-80628-z (PMC7803944; doi:10.1038/s41598-020-80628-z)
Supplement: Supplementary file 2 — Appendix I. [file 41598_2020_80628_MOESM2_ESM.docx]

Appendix 1

Character list modified from Li et al. (2014). In total, 221 morphological characters were used in the phylogenetic analysis. Character 217 was ordered and state (1) was modified as digit III almost equal to digit IV, digit II shortest. 205-220 represent characters newly added to the Clarke et al*.* (2006) dataset. Character 221 (presence of salt gland fossa) was added.

1. Premaxillae: (0) unfused in adults; (1) fused anteriorly in adults, posterior nasal [frontal] processes not fused to each other; (2) frontal processes completely fused as well as anterior premaxillae. (Ordered).
2. Premaxillary teeth: (0) present throughout premaxillae; (1) absent; (2) anteriorly reduced, posteriorly present; (3) anteriorly present, posteriorly reduced;
3. Maxillary teeth: (0) present; (1) absent.
4. Dentary teeth: (0) present; (1) absent.
5. Tooth crown serration: (0) present; (1) vestigial or absent.
6. Dentaries: (0) joined proximally by ligaments; (1) joined by bone.
7. Mandibular symphysis, two strong grooves forming an anteriorly-opening “v” in ventral view: (0) absent; (1) present.
8. Facial margin: (0) primarily formed by the maxilla, with the maxillary process of the premaxilla restricted to the anterior tip; (1) maxillary process of the premaxilla extending 1/2 facial margin; (2) maxillary process of the premaxilla extending more than 1/2 of facial margin. (Ordered).
9. Nasal [frontal] process of premaxilla: (0) short; (1) long, closely approaching frontal.
10. Nasal process of maxilla, dorsal ramus: (0) prominent, exposed medially and laterally; (1) absent or reduced to slight medial, and no lateral, exposure.
11. Nasal process of maxilla, participation of ventral ramus in anterior margin of antorbital fenestra in lateral view: (0) present, extensive; (1) small dorsal projection of the maxilla participates in the anterior margin of the antorbital fenestra, descending process of the nasals contacts premaxilla to exclude maxilla from narial margin; (2) no dorsal projection of maxilla participates in anterior margin of the antorbital fenestra. (Ordered).
12. Osseous external naris: (0) considerably smaller than the antorbital fenestra; (1) larger.
13. Ectopterygoid: (0) present; (1) absent.
14. Articulation between vomer and pterygoid: (0) present, well developed; (1) reduced, narrow process of pterygoid passes dorsally over palatine to contact vomer; (2) absent, pterygoid and vomer do not contact.
15. Palatine and pterygoid: (0) long, anteroposteriorly-overlapping, contact; (1) short, primarily dorsoventral, contact.
16. Palatine contacts: (0) maxillae only; (1) premaxillae and maxillae.
17. Vomer contacts premaxilla: (0) present; (1) absent.
18. Coronoid ossification: (0) present; (1) absent.
19. Projecting basisphenoid articulation with pterygoid: (0) present; (1) absent.
20. Basipterygoid processes: (0) long; (1) short (articulation with pterygoid sub-equal to, or longer than, amount projected from the basisphenoid rostrum).
21. Basisphenoid-pterygoid articulations: (0) located basal on basisphenoid; (1) located markedly anterior on basisphenoid (parasphenoid rostrum) such that the articulations are subadjacent on the narrow rostrum (the “rostropterygoid articulation” of Weber, 1993).
22. Basisphenoid/pterygoid articulation, orientation of contact: (0) anteroventral; (1) mediolateral; (2) entirely dorsoventral.
23. Pterygoid, articular surface for basisphenoid: (0) concave “socket,” or short groove enclosed by dorsal and ventral flanges; (1) flat to convex; (2) flat to convex facet, stalked, variably projected. (Ordered).
24. Pterygoid, kinked: (0) present, surface for basisphenoid articulation at high angle to axis of palatal process of pterygoid; (1) absent, articulation in line with axis of pterygoid.
25. Osseous interorbital septum (mesethmoid): (0) absent; (1) present.
26. Osseous interorbital septum (mesethmoid): (0) restricted to posterior or another just surpassing premaxillae/frontal contact in rostral extent does not surpass posterior edge of external nares in rostral extent; (1) extending rostral to posterior extent of frontal processes of premaxillae and rostral to posterior edge of external nares.
27. Eustachian tubes: (0) paired and lateral; (1) paired, close to cranial midline; (2) paired and adjacent on midline or single anterior opening.
28. Eustachian tubes ossified: (0) absent; (1) present.
29. Squamosal, ventral or "zygomatic” process: (0) variably elongate, dorsally enclosing otic process of the quadrate and extending anteroventrally along shaft of this bone, dorsal head of quadrate not visible in lateral view; (1) short, head of quadrate exposed in lateral view.
30. Orbital process of quadrate, pterygoid articulation: (0) pterygoid broadly overlapping medial surface of orbital process (i.e., “pterygoid ramus”); (1) restricted to anteromedial edge of process.
31. Quadrate, orbital process: (0) pterygoid articulates with anterior-most tip; (1) pterygoid articulation does not reach tip; (2) pterygoid articulation with no extent up orbital process, restricted to quadrate corpus. (Ordered).
32. Quadrate/ pterygoid contact: (0) as a facet, variably with slight anteromedial projection cradling base; (1) condylar, with a well-projected tubercle on the quadrate.
33. Quadrate, well-developed tubercle on anterior surface of dorsal process: (0) absent; (1) present.
34. Quadrate, quadratojugal articulation: (0) overlapping; (1) peg and socket articulation.
35. Quadrate, dorsal process, articulation: (0) with squamosal only; (1) with squamosal and prootic.
36. Quadrate, dorsal process, development of intercotylar incisure between prootic and squamosal cotylae: (0) absent, articular surfaces not differentiated; (1) two distinct articular facets, incisure not developed; (2) incisure present, “double headed.”
37. Quadrate, mandibular articulation: (0) bicondylar articulation with mandible; (1) tricondylar articulation, additional posterior condyle or broad surface.
38. Quadrate, pneumaticity: (0) absent; (1) present.
39. Quadrate, cluster of pneumatic foramina on posterior surface of the tip of dorsal process: (0) absent; (1) present.
40. Quadrate, pneumatization, large, single pneumatic foramen: (0) absent; (1) posteromedial surface of corpus.
41. Articular pneumaticity: (0) absent; (1) present.
42. Dentary strongly forked posteriorly: (0) unforked, or with a weakly developed dorsal ramus; (1) strongly forked with the dorsal and ventral rami approximately equal in posterior extent.
43. Splenial, anterior extent: (0) splenial stops well posterior to mandibular symphysis; (1) extending to mandibular symphysis, though non-contacting; (2) extending to proximal tip of mandible, contacting on midline.
44. Mandibular symphysis, anteroposteriorly extensive, flat to convex, dorsal-facing surface developed: (0) absent, concave; (1) flat surface developed.
45. Mandibular symphysis, symphysial foramina: (0) absent; (1) present.
46. Mandibular symphysis, symphysial foramen/foramina: (0) single; (1) paired.
47. Mandibular symphysis, symphysial foramen/foramina: (0) opening on posterior edge of symphysis; (1) opening on dorsal surface of symphysis.
48. Meckel’s groove: (0) not completely covered by splenial, deep and conspicuous medially; (1) covered by splenial, not exposed medially.
49. Anterior external mandibular fenestra: (0) absent; (1) present.
50. Jugal/postorbital contact: (0) present, (1) absent.
51. Frontal/parietal suture (0) open; (1) fused.
52. Cervical vertebrae: (0) variably dorsoventrally compressed, amphicoelous ("biconcave": flat to concave articular surfaces); (1) anterior surface heterocoelous (i.e., mediolaterally concave, dorsoventrally convex), posterior surface flat; (2) heterocoelous anterior (i.e., mediolaterally concave, dorsoventrally convex) and posterior (i.e., mediolaterally convex, dorsoventrally concave) surfaces. (Ordered).
53. Thoracic vertebrae (with ribs articulating with the sternum), one or more with prominent hypapophyses: (0) absent; (1) present. (This character does not address the presence of hypapophyses on transitional vertebrae, or “cervicothoracics”, that do not have associated ribs that articulate with the sternum [e.g., Gauthier, 1986; Chiappe, 1996]. In contrast, in Aves, well-developed hypapophyses are developed well into the thoracic series, on vertebrae with ribs articulating with the sternum.)
54. Thoracic vertebrae, count: (0) 12 or more; (1) 11; (2) 10 or fewer. (Ordered).
55. Thoracic vertebrae: (0) at least part of series with subround, central articular surfaces (e.g., amphicoelous/opisthocoelous) that lack the dorsoventral compression seen in heterocoelous vertebrae; (1) series completely heterocoelous.
56. Thoracic vertebrae, parapophyses: (0) rostral to transverse processes; (1) directly ventral to transverse processes (close to midpoint of vertebrae).
57. Thoracic vertebrae, centra, length, and midpoint width: (0) approximately equal in length and midpoint width; (1) length markedly greater than midpoint width.
58. Thoracic vertebrae, lateral surfaces of centra: (0) flat to slightly depressed; (1) deep, emarginate fossae; (2) central ovoid foramina.
59. Thoracic vertebrae with ossified connective tissue bridging transverse processes: (0) absent; (1) present.
60. Notarium: (0) absent; (1) present.
61. Sacral vertebrae, number ankylosed: (0) less than 7; (1) 7; (2) 8; (3) 9; (4) 10; (5) 11 or more; (6) 15 or more (Chiappe, 1996). (Ordered).
62. Sacral vertebrae, series of short vertebrae, with dorsally-directed parapophyses just anterior to the acetabulum: (0) absent; (1) present, three such vertebrae; (2) present, four such vertebrae. (Ordered).
63. Free caudal vertebrae, number: (0) more than 8; (1) 8 or less.
64. Caudal vertebrae, chevrons, fused on at least one anterior caudal: (0) present; (1) absent.
65. Free caudals; length of transverse processes: (0) subequal to width of centrum; (1) significantly shorter than centrum width.
66. Anterior free caudal vertebrae: (0) elongate pre/post-zygapophyses; (1) pre- and post-zygapophyses short and variably non-contacting; (2) prezygapophyses clasping the posterior surface of neural arch of preceding vertebra, postzygapophyses negligible. (Ordered).
67. Distal caudals: (0) unfused; (1) fused.
68. Fused distal caudals, morphology: (0) fused element length equal or greater than 4 free caudal vertebrae; (1) length less than 4 caudal vertebrae; (2) less than 2 caudal vertebrae in length. (Ordered).
69. Ossified uncinate processes: (0) absent; (1) present and unfused to ribs; (2) fused to ribs. (Ordered).
70. Gastralia: (0) present; (1) absent.
71. Ossified sternal plates: (0) unfused; (1) fused, flat; (2) fused, with slightly raised midline ridge; (3) fused with projected carina. (Ordered).
72. Carina or midline ridge: (0) restricted to posterior half of sternum; (1) approaches anterior limit of sternum.
73. Sternum, dorsal surface, pneumatic foramen (or foramina): (0) absent; (1) present.
74. Sternum, pneumatic foramina in the depressions (loculi costalis; Baumel and Witmer, 1993) between rib articulations (processi articularis sternocostalis; Baumel and Witmer [1993]): (0) absent; (1) present.
75. Sternum, coracoidal sulci spacing on anterior edge: (0) widely-separated mediolaterally, (1) adjacent; (2) crossed on midline.
76. Sternum, number of processes for articulation with the sternal ribs: (0) three; (1) four, (2) five; (3) six; (4) seven or more. (Ordered).
77. Sternum: raised, paired intermuscular ridges (linea intermuscularis; Baumel and Witmer, 1993) parallel to sternal midline: (0) absent; (1) present.
78. Sternum, posterior margin, distinct posteriorly projected medial and/or lateral processes: (0) absent (directly laterally projected zyphoid processes developed but not considered homologues as these are copresent with the posterior processes in the new clade); (1) with distinct posterior processes; (2) midpoint of posterior sternal margin connected to medial posterior processes to enclose paired fenestra. (Ordered).
79. Clavicles: (0) fused; (1) unfused.
80. Interclavicular angle (clavicles elongate): (0) greater than, or equal, to 90 degrees; (1) less than 90 degrees.
81. Furcula, hypocleideum: (0) absent; (1) a tubercle; (2) an elongate process. (Ordered).
82. Furcula, laterally excavated: (0) absent; (1) present.
83. Furcula, dorsal (omal) tip: (0) flat or blunt tip; (1) with a pronounced posteriorly pointed tip.
84. Furcula, ventral margin of apophysis: (0) curved, angling; (1) with a truncate or squared base.
85. Scapula and coracoid: (0) fused; (1) unfused.
86. Scapula and coracoid articulation: (0) pit-shaped scapular cotyla developed on the coracoid, and coracoidal tubercle developed on the scapula (“ball and socket” articulation); (1) scapular articular surface of coracoid convex; (2) flat.
87. Coracoid, procoracoid process: (0) absent; (1) present.
88. Coracoid: (0) height approximately equal mediolateral dimension; (1) height more than twice width, coracoid “strut-like.”
89. Coracoid, lateral margin: (0) straight to slightly concave; (1) convex.
90. Coracoid, dorsal surface (= posterior surface of basal maniraptoran theropods): (0) strongly concave; (1) flat to convex.
91. Coracoid, pneumatized: (0) absent; (1) present.
92. Coracoid, pneumatic foramen: (0) proximal; (1) distal.
93. Coracoid, lateral process: (0) absent; (1) present.
94. Coracoid, ventral surface, lateral intermuscular line or ridge: (0) absent; (1) present.
95. Coracoid, glenoid facet: (0) dorsal to, or at approximately same level as, acrocoracoid process/ “biceps tubercle”; (1) ventral to acrocoracoid process.
96. Coracoid, acrocoracoid: (0) straight; (1) hooked medially.
97. Coracoid, n. supracoracoideus passes through coracoid: (0) present; (1) absent.
98. Coracoid, medial surface, area of the foramen n. supracoracoideus (when developed): (0) strongly depressed; (1) flat to convex.
99. Angle between coracoid and scapula at glenoid: (0) more than 90 degrees; (1) 90 degrees or less.1 probably but see orientation of scapular cotyla.
100. Scapula, posterior end: (0) wider or approximately the same width as proximal dorsoventral shaft width; (1) tapering distally.
101. Scapula: (0) straight, (1) dorsoventrally curved.
102. Scapula, length: (0) shorter than humerus, (1) as long as or longer than the humerus.
103. Scapula, acromion process: (0) projected anteriorly to surpass the articular surface for coracoid (facies articularis coracoidea; Baumel and Witmer, 1993); (1) projected less anteriorly than the articular surface for coracoid.
104. Scapula, acromion process: (0) straight; (1) laterally hooked tip.
105. Humerus and ulna, length: (0) humerus longer than ulna; (1) ulna and humerus approximately the same length; (2) ulna significantly longer than humerus. (Ordered).
106. Humerus, proximal end, head in anterior or posterior view: (0) strap-like, articular surface flat, no proximal midline convexity; (1) head domed proximally.
107. Humerus, proximal end, proximal projection: (0) dorsal edge projected farthest; (1) midline projected farthest.
108. Humerus, ventral tubercle and capital incisure: (0) absent; (1) present.
109. Humerus, capital incisure: (0) an open groove; (1) closed by tubercle associated with a muscle insertion just distal to humeral head.
110. Humerus, anterior surface, well-developed fossa on midline making proximal articular surface appear v-shaped in proximal view: (0) absent; (1) present.
111. Humerus, “transverse groove”: (0) absent; (1) present, developed as a discreet, depressed scar on the proximal surface of the bicipital crest or as a slight transverse groove.
112. Humerus, deltopectoral crest: (0) projected dorsally (in line with the long axis of humeral head); (1) projected anteriorly.
113. Humerus, deltopectoral crest: (0) less than shaft width; (1) same width; (2) dorsoventral width greater than shaft width. (Ordered).
114. Humerus, deltopectoral crest, proximoposterior surface: (0) flat to convex; (1) concave.
115. Humerus, deltopectoral crest: (0) not perforate; (1) with a large fenestra.
116. Humerus, bicipital crest, pit-shaped scar/fossa for muscular attachment on anterodistal, distal or posterodistal surface of crest: (0) absent; (1) present.
117. Humerus, bicipital crest, pit-shaped fossa for muscular attachment: (0) anterodistal on bicipital crest; (1) directly ventrodistal at tip of bicipital crest; (2) posterodistal, variably developed as a fossa.
118. Humerus, bicipital crest: (0) little or no anterior projection; (1) developed as an anterior projection relative to shaft surface in ventral view; (2) hypertrophied, rounded tumescence. (Ordered).
119. Humerus, proximal end, one or more pneumatic foramina: (0) absent; (1) present.
120. Humerus, distal condyles: (0) developed distally; (1) developed on anterior surface of humerus.
121. Humerus, long axis of dorsal condyle: (0) at low angle to humeral axis, proximodistally oriented; (1) at high angle to humeral axis, almost transversely oriented.
122. Humerus, distal condyles: (0) sub-round, bulbous; (1) weakly defined, “strap-like.”
123. Humerus, distal margin: (0) approximately perpendicular to long axis of humeral shaft; (1) ventrodistal margin projected significantly distal to dorsodistal margin, distal margin angling strongly ventrally (sometimes described as a well-projected flexor process).
124. Humerus, distal end, compressed anteroposteriorly and flared dorsoventrally: (0) absent; (1) present.
125. Humerus, brachial fossa: (0) absent; (1) present, developed as a flat scar or as a scar-impressed fossa.
126. Humerus, ventral condyle: (0) length of long axis of condyle less than the same measure of the dorsal condyle; (1) same or greater.
127. Humerus, demarcation of muscle origins (e.g., m. extensor metacarpi radialis in Aves) on the dorsal edge of the distal humerus: (0) no indication of origin as a scar, a pit, or a tubercle; (1) indication as a pit-shaped scar or as a variably projected scar-bearing tubercle or facet.
128. Humerus, distal end, posterior surface, groove for passage of m. scapulotriceps: (0) absent; (1) present.
129. Humerus, m. humerotricipitalis groove: (0) absent; (1) present as a ventral depression contiguous with the olecranon fossa.
130. Ulna, cotylae: (0) dorsoventrally adjacent; (1) widely separated by a deep groove.
131. Ulna, dorsal cotyla convex: (0) absent; (1) present.
132. Ulna, distal end, dorsal condyle, dorsal trochlear surface developed as a semilunate ridge: (0) absent; (1) present.
133. Ulna, distal end, dorsal condyle, dorsal trochlear surface, extent along posterior margin: (0) less than transverse measure of dorsal trochlear surface; (1) approximately equal in extent.
134. Ulna, bicipital scar: (0) absent; (1) developed as a slightly-raised scar; (2) developed as a conspicuous tubercle.
135. Ulna, brachial scar: (0) absent; (1) present.
136. Radius, ventroposterior surface: (0) smooth; (1) with muscle impression along most of surface; (2) deep longitudinal groove.
137. Ulnare: (0) absent; (1) present.
138. Ulnare: (0) “heart-shaped,” little differentiation into short dorsal and ventral rami; (1) V-shaped, well-developed dorsal and ventral rami.
139. Ulnare, ventral ramus (crus longus, Baumel and Witmer, 1993): (0) shorter than dorsal ramus (crus brevis); (1) same length as dorsal ramus; (2) longer than dorsal ramus.
140. Semilunate carpal and metacarpals: (0) no fusion; (1) incomplete proximal fusion; (2) complete proximal fusion; (3) complete proximal and distal fusion. (Ordered).
141. Semilunate carpal, position relative to metacarpal I: (0) over 1/2 or more of proximal surface; (1) over less than 1/2 proximal surface. (Ordered)
142. Metacarpal III, anteroposterior diameter as a percent of same dimension of metacarpal II: (0) approximately equal or greater than 50%; (1) less than 50%.
143. Metacarpal I, anteroproximally-projected muscular process: (0) absent no distinct process visible; (1) small knob at anteroproximal tip of metacarpal; (2) tip of process just surpasses the distal articular facet for phalanx 1 in anterior extent; (3) tip of extensor process conspicuously surpasses articular facet by approximately half the width of facet, producing a pronounced knob; (4) tip of extensor process conspicuously surpasses articular facet by approximately the width of facet, producing a pronounced knob. (Ordered).
144. Metacarpal I, anterior surface: (0) roughly hourglass-shaped proximally, at least moderately expanded anteroposteriorly, and constricted just before flare of articulation for phalanx 1; (1) anterior surface broadly convex.
145. Metacarpal I, distal articulation with phalanx I: (0) ginglymoid; (1) shelf.
146. Pisiform process: (0) absent; (1) present.
147. Carpometacarpus, ventral surface, supratrochlear fossa deeply excavating proximal surface of pisiform process: (0) absent; (1) present.
148. Intermetacarpal space (between metacarpals II and III), (0) reaches proximally as far as the distal end of metacarpal I; (1) terminates distal to end of metacarpal I.
149. Carpometacarpus, distal end, metacarpals II and III, articular surfaces for digits: (0) metacarpal II sub-equal or surpasses metacarpal III in distal extent; (1) metacarpal III extends farther.
150. Intermetacarpal process or tubercle: (0) absent; (1) present as scar; (2) present as tubercle or flange. (Ordered).
151. Manual digit II, phalanx 1: (0) subcylindrical to subtriangular; (1) strongly dorsoventrally compressed, flat caudal surface.
152. Manual digit II, phalanges: (0) length of phalanx II-1 less than or equal to that of II-2; (1) longer.
153. Manual digit II, phalanx 2, "internal index process" (Stegmann, 1978) on posterodistal edge: (0) absent; (1) present (Clarke and Chiappe, 2001).
154. Ilium, ischium, pubis, proximal contact in adult: (0) unfused; (1) partial fusion (pubis not ankylosed); (2) completely fused. (Ordered).
155. Ilium/ischium, distal coossification to completely enclose the ilioischiadic fenestra: (0) absent; (1) present.
156. Ischium: (0) forked (dorsal process present); (1) straight, no dorsal process.
157. Ischium, dorsal process: (0) does not contact ilium; (1) contacts ilium.
158. Ischium and pubis: (0) not subparallel, pubis directed ventrally; (1) subparallel, pubis posteriorly directed; (2) pubis appressed to ischium. (Ordered).
159. Laterally projected process on ischiadic peduncle (antitrochanter): (0) directly posterior to acetabulum; (1) posterodorsal to acetabulum.
160. Preacetabular pectineal process (Baumel and Witmer, 1993): (0) absent; (1) present as a small flange; (2) present as a well-projected flange. (Ordered).
161. Preacetabular ilium: (0) approach on midline, open, or cartilaginous connection; (1) coossified, dorsal closure of “iliosynsacral canals”.
162. Preacetabular ilium extends anterior to first sacral vertebrae: (0) no free ribs overlapped; (1) one or more ribs overlapped.
163. Postacetabular ilium: (0) dorsoventrally oriented; (1) mediolaterally oriented.
164. Postacetabular ilium, ventral surface, renal fossa developed: (0) absent; (1) present.
165. Ilium, m. cuppedicus fossa as broad, mediolaterally-oriented surface directly anteroventral to acetabulum: (0) present; (1) surface absent, insertion variably marked by a small entirely lateral fossa anterior to acetabulum.
166. Ischium, posterior demarcation of the obturator foramen: (0) absent; (1) present, developed as a small flange or raised scar contacting / fused with pubis and demarcating the obturator foramen distally.
167. Ischium, length relative to that of pubis: (0) 1/3 or greater total pubis length extends posterior to end of ishium; (1) less than 1/3 pubis extends farther than end of ishium.
168. Pubis: (0) sub-oval in cross section; (1) compressed mediolaterally.
169. Pubes, distal contact: (0) contacting, variably coossified into symphysis; (1) non-contacting.
170. Distal end of pubis: (0) expanded, flared; (1) straight, subequal, in proportion with rest of pubis.
171. Femur, fossa for insertion of lig. capitis femoris: (0) absent; (1) present.
172. Femur, posterior trochanter: (0) present, developed as a slightly projected tubercle or flange; (1) hypertrophied, “shelf-like” conformation (in combination with development of the trochanteric shelf; see Hutchinson, 2001); (2) absent (Chiappe, 1991). (Ordered).
173. Femur, lesser and greater trochanters: (0) separated by a notch; (1) developed as a single trochanteric crest.
174. Femur, patellar groove: (0) absent; (1) present.
175. Femur: (0) ectocondylar tubercle and lateral condyle separated by deep notch; (1) ectocondylar tubercle and lateral condyle form single trochlear surface.
176. Femur, posterior projection of the lateral border of the distal end, continuous with lateral condyle: (0) absent; (1) present.
177. Laterally-projected fibular trochlea: (0) absent; (1) present, developed as small notch; (2) a shelf-like projection. (Ordered).
178. Femur, popliteal fossa: (0) a groove open distally and bounded medially and laterally by narrow condyles; (1) closed distally by expansion of both condyles (primarily the medial).
179. Calcaneum and astragalus: (0) unfused to each other or tibia in adult; (1) fused to each other, unfused to tibia; (2) complete fused to each other and tibia. (Ordered).
180. Tibia, cnemial crest(s): (0) lateral crest only; (1) lateral and anterior crests developed.
181. Tibia/tarsal formed condyles: (0) medial condyle projecting farther anteriorly than lateral; (1) equal in anterior projection.
182. Tibia/tarsal formed condyles, extensor canal: (0) absent; (1) an emarginate groove; (2) groove bridged by an ossified supratendinal bridge. (Ordered).
183. Tibia/tarsal formed condyles, tuberositas retinaculi extensoris (Baumel and Witmer, 1993) indicated by short medial ridge or tubercle proximal to the condyles close to the midline and a more proximal second ridge on the medial edge: (0) absent; (1) present.
184. Tibia/tarsal formed condyles, mediolateral widths: (0) medial condyle wider; (1) approximately equal; (2) lateral condyle wider. (Ordered).
185. Tibia/tarsal formed condyles: (0) gradual sloping medial constriction of condyles; (1) no medial tapering of either condyle.
186. Tibia/tarsal formed condyles, intercondylar groove: (0) mediolaterally broad, approximately 1/3 width of anterior surface; (1) less than 1/3 width of total anterior surface.
187. Tibia, extension of articular surface for distal tarsals/tarsometatarsus: (0) no posterior extension of trochlear surface, or restricted to distal-most edge of posterior surface; (1) well-developed posterior extension, sulcus cartilaginis tibialis of Aves (Baumel and Witmer, 1993), distinct surface extending up the posterior surface of the tibiotarsus; (2) with well-developed, posteriorly projecting, medial and lateral crests. (Ordered).
188. Tibia, distal-most mediolateral width: (0) wider than mid-point of shaft, giving distal profile a weakly developed triangular form; (1) approximately equal to shaft width, no distal expansion of whole shaft, although condyles may be variably splayed mediolaterally.
189. Fibula: (0) reaches tarsal joint articulating into distinct socket formed between the proximal tarsals and the tibia; (1) reduced in length, does not reach tarsal joint.
190. Distal tarsals and metatarsals, fusion: (0) distal tarsals fuse to metatarsals; (1) distal tarsals fuse to metatarsals and proximal metatarsals coossify; (2) distal tarsals fuse to metatarsals, and metatarsals fuse to each other proximally and distally; (3) extreme distal fusion, distal vascular foramen closed (Martin, 1983; Cracraft, 1986). (Ordered)
191. Metatarsal V: (0) present; (1) absent.
192. Metatarsal III: (0) proximally in plane with II and IV; (1) proximally displaced plantarly, relative to metatarsals II and IV.
193. Tarsometatarsus, intercotylar eminence: (0) absent; (1) well developed, globose.
194. Tarsometatarsus, projected surface or grooves on proximoposterior surface (associated with the passage of tendons of the pes flexors in Aves; hypotarsus): (0) absent; (1) developed as posterior projection with flat posterior surface; (2) projection, with distinct crests and grooves; (3) at least one groove enclosed by bone posteriorly. (Ordered).
195. Tarsometatarsus, proximal vascular foramen(foramina): (0) absent; (1) one, between metatarsals III and IV; (2) two. (Ordered).
196. Metatarsal I: (0) straight; (1) curved or distally deflected but not twisted, ventral surface convex “J shaped”; (2) deflected and twisted such that the ventromedial surface is concave proximal to trochlear surface for phalanx I. (Ordered).
197. Metatarsal II tubercle (associated with the insertion of the tendon of the m. tibialis cranialis in Aves): (0) absent; (1) present, on approximately the center of the proximodorsal surface of metatarsal II; (2) present, developed on lateral surface of metatarsal II, at contact with metatarsal III or on lateral edge of metatarsal III. (Ordered).
198. Metatarsal II, distal plantar surface, fossa for metatarsal I (fossa metatarsi I; Baumel and Witmer, 1993): (0) absent; (1) shallow notch; (2) conspicuous ovoid fossa. (Ordered).
199. Metatarsal II, articular surface for first phalanx: (0) ginglymoid; (1) rounded.
200. Metatarsals, relative mediolateral width : (0) metatarsal IV approximately the same width as metatarsals II and III; (1) metatarsal IV narrower than MII and MIII; (2) metatarsal IV greater in width than either metatarsal II or III.
201. Metatarsals, comparative trochlear width: (0) II approximately the same size as III and/or IV; (1) II wider than III and/or IV; (2) II narrower than III and/or IV.
202. Distal vascular foramen: (0) simple, with one exit; (1) forked, two exits (plantar and distal) between metatarsals III and IV.
203. Metatarsal III, trochlea in plantar view, proximal extent of lateral and medial edges of trochlea: (0) absent, trochlear edges approximately equal in proximal extent; (1) present, lateral edge extends farther.
204. Metatarsal II, distal extent of metatarsal II relative to metatarsal IV: (0) approximately equal in distal extent; (1) metatarsal II shorter than metatarsal IV, but reaching distally farther than base of metatarsal IV trochlea; (2) metatarsal II shorter than metatarsal IV, reaching distally only as far as base of metatarsal IV trochlea. (Ordered).
205. Predentary element: (0) absent; (1) present.
206. Pygostyle, well developed transverse processes throughout length of pygostyle: (0) absent; (1) present.
207. Sternum: distal-most expansion or flare of the posterolateral process: (0) absent, tip of process subequal in width with the more proximal part of the process; (1) present, strongly flared, significantly broader than the more proximal part of the process.
208. Sternum, xiphoid process: (0) present; (1) absent.
209. Sternal incisures, anterior extent relative to rib articulations: (0) terminate sub-equal with posterior-most rib facet; (1) terminate well posterior to the posterior-most rib facet.
210. Sternal carina, location of apex: (0) posterior to anterior margin; (1) equal or surpassing anterior margin.
211. Coracoid, width at the sternal articulation: (0) broad flares markedly; (1) very narrow.
212. Coracoid, glenoid facet, position relative to the scapular cotyla: (0) extends to sternal margin of the cotyla in lateral view; (1) over half of the glenoid facet lies omal to the cotyla in lateral view.
213. Manual digit I, distal extent relative to metacarpal II (including ungual, I:2 if present): (0) digit I projects distal to distal end of metacarpal II; (1) terminates close to the terminus of metacarpal II; (2) over half the length of the metacarpal II but does not reach its distal end; (3) equal to, or less, than one half of the length of metacarpal II.
214. Manual phalanx III:1,flexor tubercle: (0) absent or indistinct, not developed as a tubercle; (1) present .
215. Manual digit III, number of phalanges: (0) four; (1) two; (2) one; (3) three.
216. Ilium, length of preacetabular ilium compared to postactabular ilium: (0) preactabular longer; (1) equal; (2) postacetabular ilium longer. (Ordered).
217. Pedal digits, relative length: (0) digit III longest, followed by digit IV, digit II shortest; (1) digit III almost equal to digit IV, digit II shortest; (2) digit IV longest, then digit III, digit II shortest. (Ordered)
218. Hallux, claw to phalanx proportions, 1:1: (0) equal to or longer; (1) shorter.
219. Pedal digit II, proportions of II:3 to II:1: (0) II: 3 subequal to or longer than II:1 and; (1) II:3 shorter.
220. Tail feathers, number of associated with free caudals: (0) more than 14, (1) none, but two associated with pygostyle; (2) none, but more than two associated with pygostyle.

221. Supraorbital salt gland fossa: (0) absent; (1) present, but shallow; (2) present, deep.

Appendix S1. The data matrix utilized in the phylogenetic analysis. Please see the attached file.
